# Complex Kinetic Models Predict β‑Carotene Production and Reveal Flux Limitations in Recombinant *Saccharomyces cerevisiae* Strains

**DOI:** 10.1021/acssynbio.5c00256

**Published:** 2025-09-02

**Authors:** Benjamín R. Elizondo, Pedro A. Saa

**Affiliations:** † Departamento de Ingeniería Química y Bioprocesos, Escuela de Ingeniería, 28033 Pontificia Universidad Católica de Chile, Santiago 7820436, Chile; ‡ Instituto de Ingeniería Matemática y Computacional, Pontificia Universidad Católica de Chile, Santiago 7820436, Chile

**Keywords:** kinetic model, Saccharomyces cerevisiae, beta-carotene, mevalonate pathway, metabolic control analysis, metabolic engineering

## Abstract

β-Carotene is a high-value compound with multiple commercial applications as a pigment and due to its antioxidant properties. For its industrial production, precision fermentation using engineered microorganisms has been proposed as an attractive alternative given consumer concerns and technical limitations of traditional production methods such as chemical synthesis and extraction from plants. However, the factors limiting microbial production are complex and remain poorly understood, hindering bioprocess scale-up. To tackle this limitation, we built and evaluated kinetic model ensembles of the native mevalonate and the heterologous β-carotene production pathways in recombinant *Saccharomyces cerevisiae* strains to identify bottlenecks limiting the production flux. For this task, flux and transcriptomic data from chemostat cultivations were generated and combined with literature information for simulating model structures capturing different degrees of kinetic detail and complexity within the ABC-GRASP framework. Our results showed that detailed kinetic models including both allosteric regulation and complex mechanistic descriptions (e.g., enzyme promiscuity) are necessary to explain the metabolic phenotype of recombinant strains in different conditions. Calculation of flux and concentration response coefficients of the detailed models revealed that the promiscuous CrtYB enzyme exerts the highest control over β-carotene production at different growth rates in the best producer. Simulation of various enzyme and metabolite perturbations confirmed the above result and discarded other seemingly intuitive targets for intervention, e.g., upregulation of ERG10. Overall, this work deepens our understanding about the factors limiting β-carotene production in yeast, providing mechanistic models for *in silico* metabolic prospection and rational design of genetic interventions.

## Graphical abstract

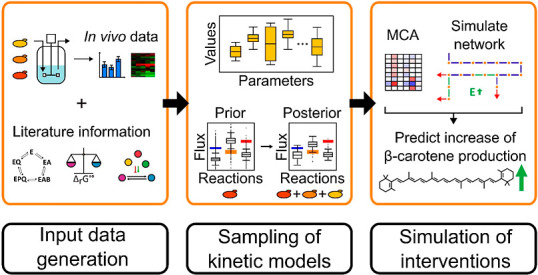

## 1 Introduction

β-Carotene is a red-orange natural pigment of high commercial value used in the food, pharmaceutical, cosmetic, and even textile industries.^1^ This metabolic compound belongs to the group of carotenoids with at least one unsubstituted β-ring and is necessary for the production of retinoid and vitamin A in humans.^2,3^ Due to its antioxidant properties, its consumption supports eye fitness in humans,^4^ reduces the symptoms of Alzheimer’s disease,^5,6^ protects against gastric cancer,^7^ and helps maintaining a healthy skin.^8^ Commercial β-carotene can be produced by extraction from natural sources, chemical synthesis, and microbial biosynthesis.^3^ While this carotenoid is present in high concentrations in most plants, its extraction is challenging due to the low yields, biomass waste, high processing costs, and lack of color standardization.^1^ On the other hand, chemical synthesis overcomes most of these issues but suffers from a diminished antitumoral^9^ or even carcinogenic^10^ effects, and it is often rejected by consumers.^11^ Microbial β-carotene bioproduction has emerged as a viable alternative, addressing many of the previous challenges associated with its natural origin and scalability potential.^1^ Although microorganisms such as *Dunaliella Bandawil*, *Spirulina* and *Blakeslea Trispora* naturally produce this compound,^12^ bioprocess optimization remains challenging and current titers and yields are still not competitive.^11^

Metabolic engineering of biotechnological workhorses like *Escherichia coli* and yeast has been pointed as an attractive approach for improving β-carotene bioproduction.^13^ Heterologous genes have been integrated into *E. coli, Yarrowia lipolytica*, and *Saccharomyces cerevisiae* for β-carotene accumulation.^11^ Complementary strategies have been also implemented to further increase production titers, namely: enzyme engineering, increasing the precursors and cofactors supply, cellular membrane modification, and fine-tuned gene expression.^14^ Despite these advances, strain development remains complex and time-consuming, requiring various iterations for reaching high β-carotene productivity and yields.^1^

Model-driven metabolic engineering approaches have gained increasing attention for their ability to drive experimentation by proposing more rational designs.^15^ Particularly, kinetic models are useful tools for understanding metabolic behaviors and guiding genetic interventions. Unlike constraint-based stoichiometric models,^16^ these models coherently integrate multiomics data including enzyme and metabolite concentrations, as well as thermodynamics, regulatory and kinetic information.^17–19^ However, their highly nonlinear nature, uncertain model structure, and large number of parameters render the model building task challenging due to the high parameter dimensionality and prediction uncertainty.^18,20^ Among the computational frameworks for building these models, the General Reaction and Assembly Platform (GRASP) is noticeable for proposing thermodynamically feasible and detailed kinetic models of metabolism capable of capturing the parameter uncertainty within an Approximate Bayesian Computation (ABC) setting.^21^ Unlike other approaches, the capabilities of this framework focus on the computation of metabolic states and sensitivity analysis under the Bayesian paradigm, while preserving a thermodynamically feasible and detailed kinetic description of reaction fluxes.^16,18^ Its most recent version enables the mechanistic description of allosteric regulation, substrate-level regulation, and promiscuous enzymes,^22–24^ complex kinetic features that are present in the β-carotene production pathway.

In this study, we evaluated kinetic model ensembles of the native mevalonate and the heterologous β-carotene production pathways in recombinant *S. cerevisiae* strains to identify bottlenecks limiting metabolic fluxes. For this task, flux and transcriptomic data were generated in chemostat cultivations for model training. Thermodynamic, regulatory and reaction mechanism information was collected from the literature to propose and parametrize model ensembles using GRASP^25^ that describe different model structures anchored at a reference condition. Competing model structures reflect different degrees of kinetic detail and complexity, enabling a more systematic analysis of the relevant kinetic mechanisms underpinning the functioning of the production pathway. Using an ABC rejection approach,^21^ accurate models were selected that satisfied multiple experimental conditions and then used to perform response coefficients calculations to reveal the control structure of the system. Finally, the simulation of promising genetic interventions revealed promising nontrivial metabolic engineering targets for improving β-carotene production.

## 2 Materials and Methods

The overall workflow employed in this study was composed of two main phases: biological data generation and model building (Figure 1). Experimental data obtained from recombinant β-carotene-producing *S. cerevisiae* strains grown in chemostat cultivations at different dilution rates were analyzed and integrated into different kinetic model ensembles. Gene expression, metabolite concentrations, and metabolic fluxes of the production pathway were measured or estimated, and together combined with reported thermodynamic, kinetic and regulatory information for model construction. For this task, the GRASP framework was used to evaluate models with different mathematical structures, varying degrees of kinetic detail, and metabolic regulations. Incorporation of multiple experimental steady states enabled selection of the most likely explanatory models, revealing the importance of the considered mechanisms in the model structure. Finally, a posteriori prediction of the control structure using Metabolic Control Analysis (MCA)^26,27^ yielded attractive targets for metabolic engineering that can be subsequently evaluated under different scenarios. In the following, we first present the experimental methods for biological data generation and then the computational methods for model development.

**Figure 1:**
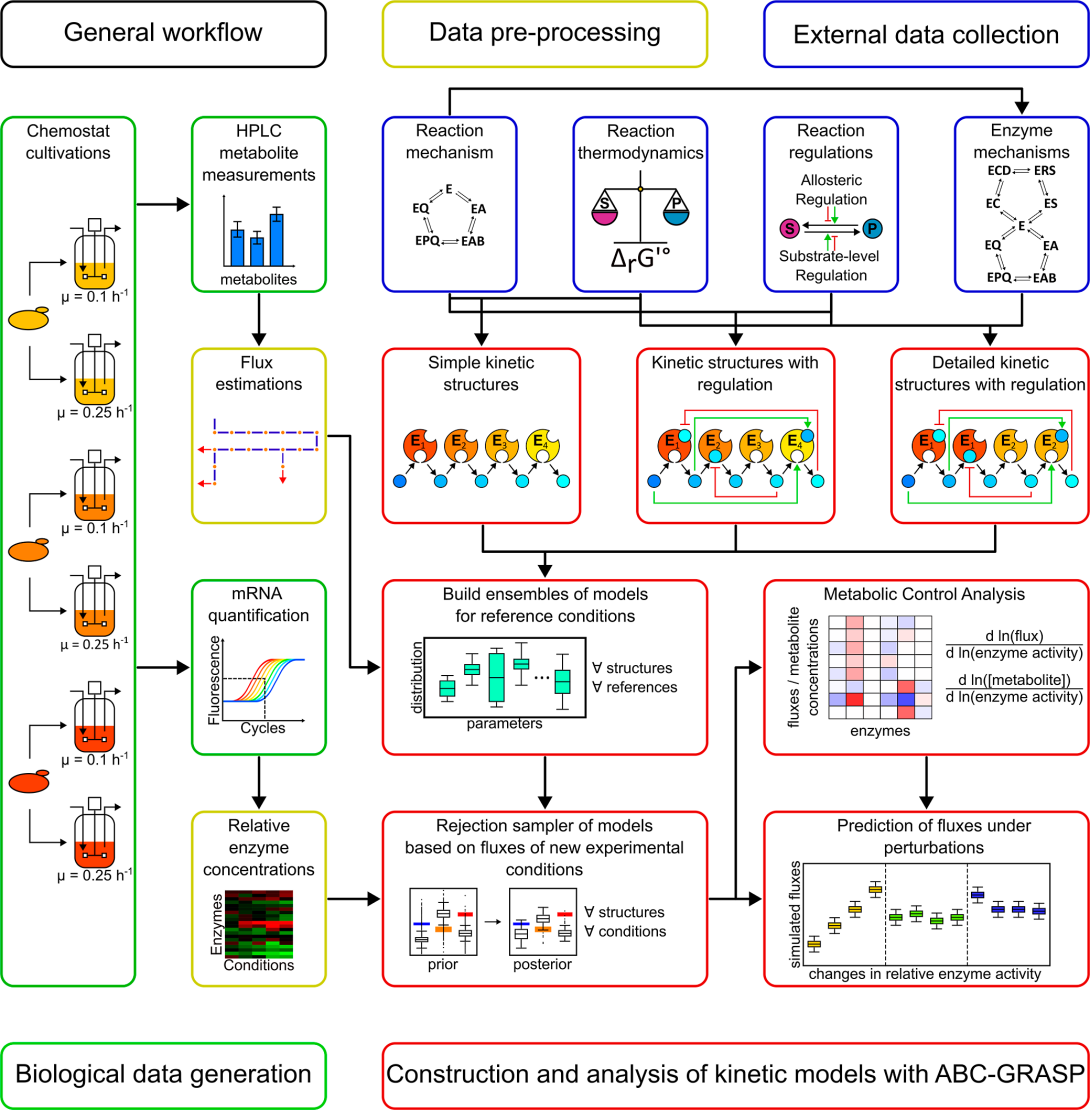
Overview of the general workflow of the study. Biological data generation (green boxes) consisted in the chemostat cultivation of three β-carotene producing *S. cerevisiae* strains at two growth rates. Key metabolites and gene transcripts of the pathway were quantified. A data preprocessing step (yellow boxes) was implemented to generate the experimental input for model generation. In the external data collection step (blue boxes), data from databases and the literature were employed to define the reaction mechanisms, regulatory features, and thermodynamic properties of the evaluated kinetic models. Finally, the construction and analysis of kinetic models employed the ABC-GRASP Bayesian framework (red boxes). Different model structures with increasing kinetic detail were developed to generate model ensembles anchored at a reference condition. These ensembles were then evaluated by assessing flux predictions under new experimental conditions, enabling the selection of more accurate models. The latter models were then employed to perform MCA calculations to identify attractive targets for metabolic engineering. Perturbations of these and additional genetic targets were simulated under different conditions, revealing promising interventions for future experimentation.

### 2.1 Biological Data Generation

#### 2.1.1 Strains and Cell Cultivations

Three *S. cerevisiae* strains constructed previously by López et al.^28^ were employed in this study. The recombinant CEN.PK2–1c-based strains β-car2.1, β-car3, and β-car4.b, from now on referred to as β-car2, β-car3, and β-car4, were selected and phenotypically characterized. These strains include the same heterologous genes CrtE, CrtYB, and CrtI from *Phaffia rhodozyma* but in different dosage (copies) in the integration sites XI-5, XI-3, and X-2, which are described in Jessop-Fabre et al.^29^ (Figure 2a). Each gene is integrated under constitutive promoters TEF1, PGK1 or TDH3 using the ADH1 or CYC1 terminators. The difference in gene copy numbers causes variations in β-carotene and lycopene accumulation, enabling observation of different production phenotypes and pathway operation conditions for subsequent model building^28^ (Figure 2b).

**Figure 2:**
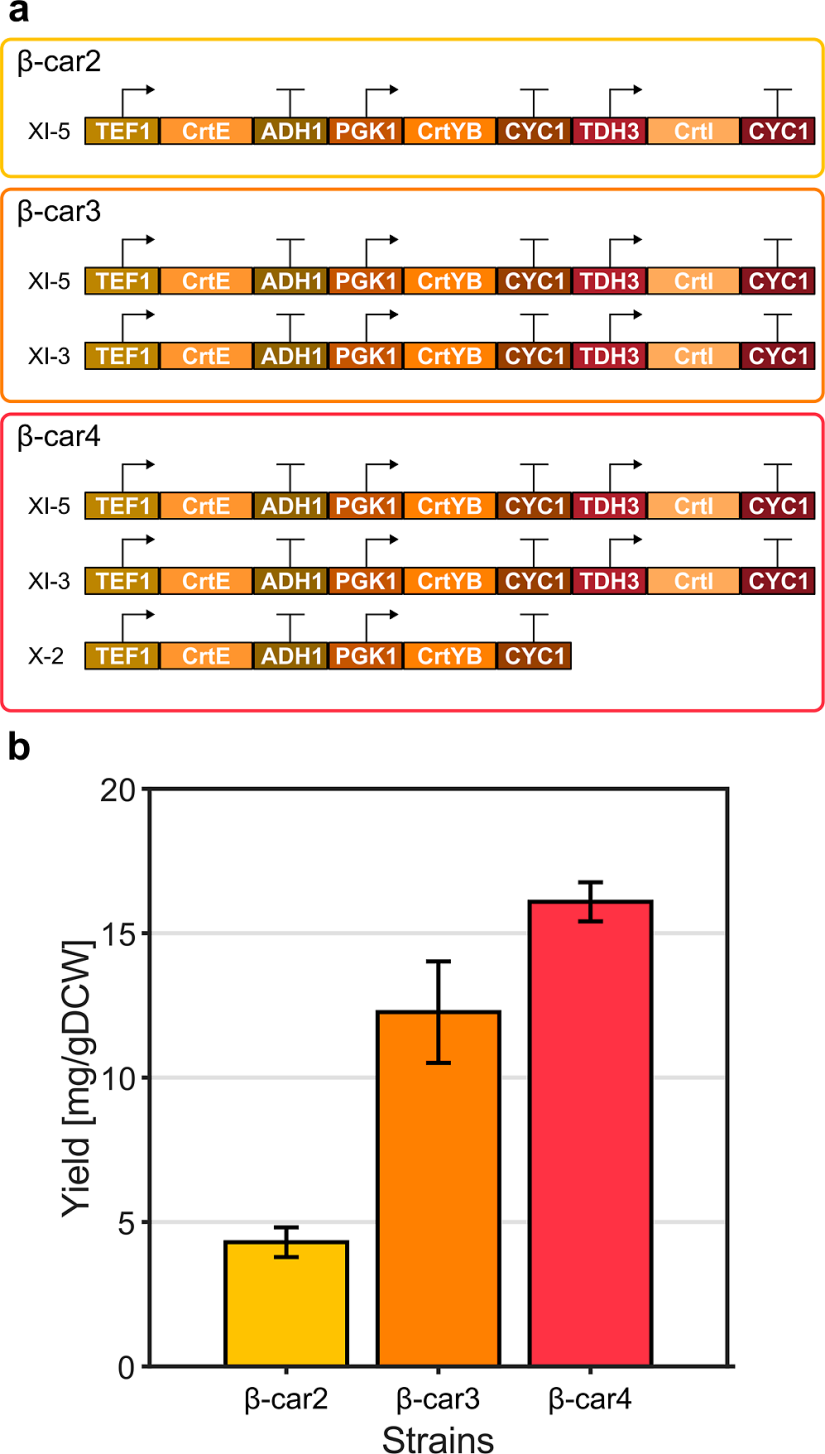
Transcriptional unit architectures and carotenoid production yields in shake flask fermentations of the recombinant *S. cerevisiae* strains analyzed in this study. (a) Schematics of the transcriptional unit architectures driving heterologous gene expression in the recombinant yeast strains, including promoters, terminators, and integration sites. (b) Total carotenoid production after 72 h (mean ± standard deviation of triplicates) of recombinant strains grown aerobically in shake flasks with YPD medium. Transcriptional unit architectures information and metabolic production data were extracted from López et al.^28^

The medium for the cultivations was the Yeast Nitrogen Base w/o AA, Carbohydrate & w/o AS (YNB) purchased from US Biologicals (MA, USA). The latter was used at 1x concentration supplemented with amino acids (histidine, leucine, tryptophan, methionine, and uracil) for the auxotrophic requirements of CEN.PK2–1c.^30^ The medium also included 12 g/L of glucose as carbon source and 5 g/L ammonium sulfate as sulfur source.^31^ The strains were cultivated in carbon-limited, aerobic stirred-tank bioreactors in continuous mode with a working volume of 500 mL. Each of the strains were grown at dilution rates of 0.1 and 0.25 h^–1^ with a fixed agitation of 200 rpm. The temperature was controlled at 30 ± 0.5 °C using a thermal-regulating water jacket. pH was kept at 5.0 by the addition of a NaOH solution 1 M. The airflow was maintained at 0.3 L/min and the dissolved oxygen concentration was above 2.7 ppm throughout the cultivation to ensure aerobiosis.

#### 2.1.2 Sampling, Extraction, and Metabolite Analysis

The cultivation broth was routinely sampled for biomass estimation using OD600 nm measurements performed in a spectrophotometer (Thermo Fisher Scientific, MA, USA). Dry cell weight was approximated based on estimated OD600/DCW conversion factors (0.53, 0.54, and 0.50 for strains β-car2, β-car3, and β-car4, respectively) (for details refer to the Supplementary Methods in the Supporting Information). Sampling was performed after 5 residence times. Intracellular carotenoids, extracellular metabolites, and intracellular RNA were sampled in technical triplicates. For carotenoid samples, 15 mL of broth were centrifuged at 13,000 RCF for 4 min. The pellet was stored at −80 °C to prevent degradation^32^ for later carotenoid extraction. On the other hand, the supernatant was stored at −20 °C for later analysis of extracellular compounds.^33^ For RNA samples, 15 mL of broth were treated with RNA Save Solution (Biological Industries, Israel) following the protocol for yeast cells recommended by the manufacturer. Isolated cells were then immediately frozen at −80 °C for later RNA extraction.

Carotenoids were extracted from each sample and then measured by High-Performance Liquid Chromatography (HPLC) using an adapted version of the protocol presented in López et al.^28^ Briefly, the sampled cell pellets were resuspended in 1 mL of hexane and then disrupted with 0.5 mL of 0.5 mm zirconia/glass beads in a homogenizer (Benchmark Scientific, NJ, United States) for 8 min at 3500 rpm. The homogenized samples were centrifuged for 2 min at 20,000 RCF and the supernatant saved in a 1.5 mL tube protected from light. Hexane addition and the homogenization step were repeated until the pellet was clear to ensure complete carotenoids extraction. The hexane was evaporated in a vacuum centrifugal concentrator (Gyrozen, South Korea) operating at 1000 rpm for 20 min. Once the liquid was removed, the traces of carotenoids in each tube were resuspended in 1 mL of acetone. All the extracts belonging to the same original sample were mixed into one. Finally, lycopene and β-carotene were separated and measured using HPLC as described by López et al.^28^ The measurement of glucose, ethanol, glycerol, and acetate in the culture media were performed by HPLC as described by Aceituno et al.^34^

#### 2.1.3 Reverse-Transcriptase Quantitative Polymerase Chain Reaction

RNA was extracted from each sample using the E.Z.N.A Total RNA Kit I (OMEGA Bio-Tek, GA, USA) following the Cultured Cell and DNase I Digestion Protocol. An additional homogenization step was required at the beginning of the extraction. The latter step consisted of disrupting the cells resuspended in the TRK Lysis Buffer from the kit in a homogenizer. Cells were disrupted with 0.5 mL of 0.5 mm RNase-free glass beads (OMEGA Bio-Tek) for 10 min at 3500 rpm. After extraction, RNA concentration and purity were quantified using Nanodrop (Thermo Fisher Scientific). Integrity and DNA contamination were checked by denaturing RNA electrophoresis as described by Masek^35^ with 0% formamide. Next, a cDNA library was generated using the AffinityScript QPCR cDNA Synthesis kit (Agilent Technologies, CA, USA) with Oligo-dT primers.

To measure the assay efficiency of the qPCR plates, standards were prepared from the cDNA of one of the retrotranscribed RNA samples. First, the target sequence of each gene was amplified by PCR using PfuUltra II Fusion HS DNA Polymerase (Agilent Technologies, CA, USA). Then, the lengths of the amplicons and the absence of off-target sequences were checked by agarose electrophoresis. Subsequently, the DNA bands were extracted from the agarose gel and purified using the E.Z.N.A. Gel Extraction Kit (OMEGA Bio-Tek) Spin Protocol. Each of the fragments was amplified once again by PCR. Finally, the DNA products from the reaction were purified utilizing the E.Z.N.A Cycle Pure Kit (OMEGA Bio-Tek) Protocol for Small Nucleic Acid Fragments.

qPCR assays were conducted in an AriaMx Real-time PCR System (Agilent Technologies, CA, USA) using the Brilliant II SYBR Green QPCR Master Mix (Agilent Technologies). Only one gene was measured per plate/assay. Three separate reactions were performed for each of the technical triplicates per condition. In addition, each triplicate had a No-RT control on the plate to account for DNA contamination. For the standard curve, each gene was serially diluted by 10^–5^ to 10^–11^ of the original titers. Three separate reactions were used for each of the dilutions. Finally, three NTC reactions were used to examine contamination of the master mix. Analysis of raw fluorometric data was performed in the Agilent AriaMx Software© (version 2.0) to obtain threshold cycles for each of the qPCR samples. Further details required to abide the MIQE guidelines^36^ are included in the Supplementary Methods in the Supporting Information.

#### 2.1.4 Estimation of Experimental Metabolic Rates

The fluxes of the metabolic pathway presented in Figure 3 were determined based on three experimentally estimated fluxes. SK_lyc (lycopene sink), SK_b_car (β-carotene sink), and ERG9b conversion of presqualene diphosphate (psql) to squalene (sql) are sufficient to characterize all the fluxes in the production pathway under steady-state. The drains of lycopene and β-carotene represent the intracellular accumulation of carotenoids in the cell membrane.^37,38^ It was assumed that the cytosolic concentrations of carotenoids were negligible compared to the content in the membrane,^39^ which is consistent with the hydrophobic nature of these compounds.^40^ In this way, the flux drains of lycopene and β-carotene can be readily estimated from the measured carotenoid profile content and biomass concentration in the broth.

**Figure 3:**
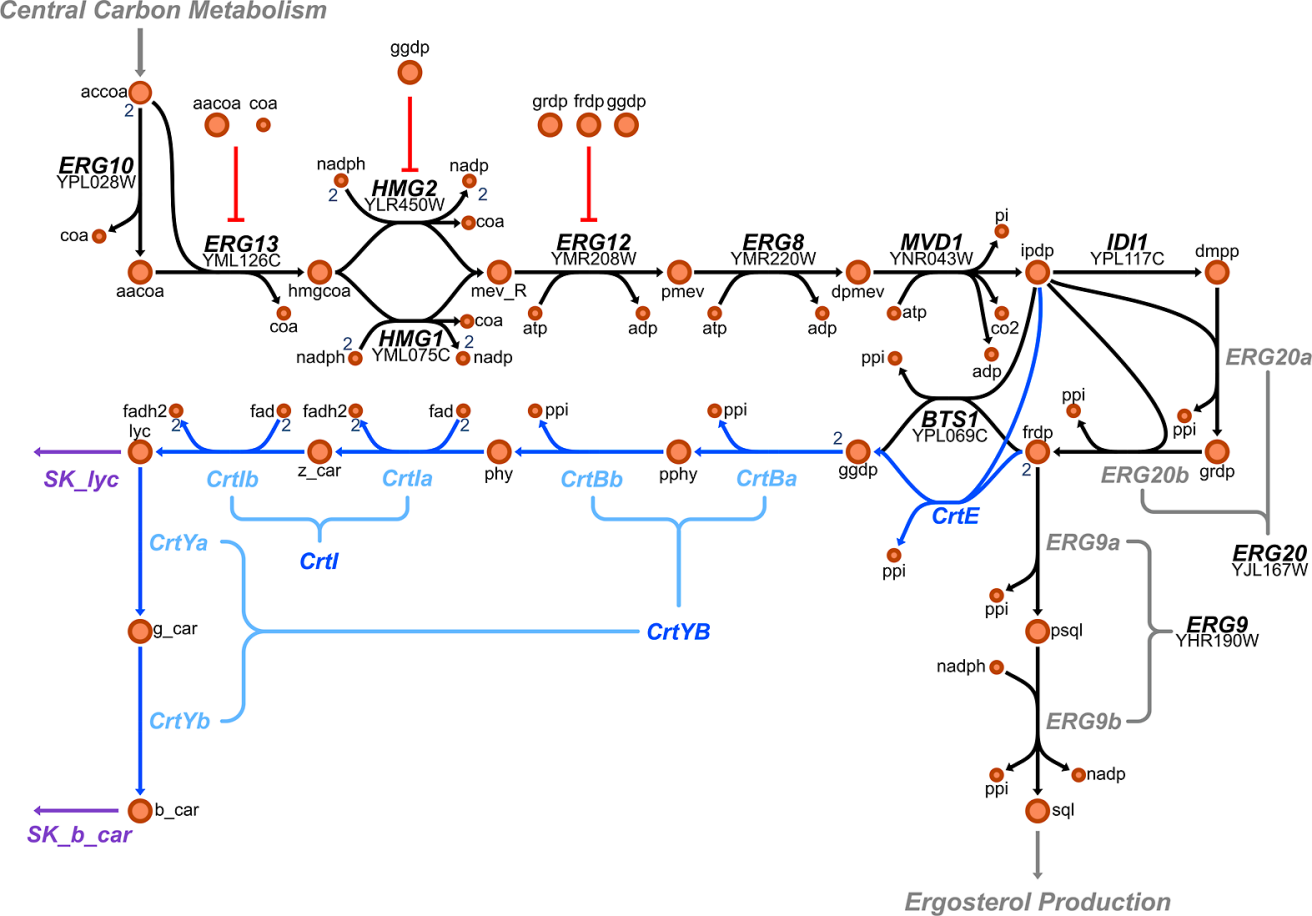
Mevalonate and β-carotene pathway model. The overall metabolic network included in the kinetic models is presented in the illustration. The model encompasses 29 metabolites, 14 enzymes and 22 reactions (fluxes). Three of these fluxes are free and were determined in each condition (SK_lyc, SK_b_car and ERG9b). Metabolic regulations (inhibitions) are shown in red. The image was created using Escher web application.^41^ Reactions and metabolites abbreviations are listed in the Supporting Information Tables S1 and S2.

The ERG9b flux was estimated using Flux Variability Analysis (FVA).^42^ For this task, the modified yeast GEnome-scale Metabolic model (GEM) presented by López^28^ was employed. Similar to this study, it was assumed that all the compounds downstream of squalene had no significant drains and that their production/consumption were contained within the ergosterol pathway. Accordingly, and considering that ergosterol is an essential metabolite for biomass formation,^43,44^ the ERG9b flux only depends on the specific growth rate, which corresponds to the dilution rate in a chemostat cultivation. The fluxes in mmol/gDCW/h were transformed to mmol/L/h assuming a cell density of 1.1029 ± 0.0026 g/mL^45^ and a moisture content of 1.525 ± 0.004 g/gDCW.^46^ Further details regarding flux calculations are included in the Supporting Information Text S4.

#### 2.1.5 Estimation of Relative Enzyme Concentrations

Relative mRNA molecule counts were used as proxies for relative enzyme concentration estimation. Previous studies indicate that, at steady state, specific gene variation as determined by mRNA levels is the primary determinant of protein abundance.^47,48^ The nonparametric Spearman rank correlation of both quantities has been reported to be above 0.5 in yeast,^47^ mRNA concentration explaining most of the observed variance in protein abundance.^48,49^ Furthermore, linear relationships between enzyme concentrations and their respective mRNA transcript levels in *S. cerevisiae* have been found for individual genes when growing at steady state in chemostats.^50,51^ Therefore, the relative transcript expression was assumed to reasonably approximate the corresponding relative enzyme concentrations. However, all conditions’ mRNA were normalized against a common reference condition on a gene-specific basis. The Common Base method^52^ was employed to calculate the relative mean mRNA concentrations and their 95% confidence intervals for each pathway gene (Supporting Information Text S5).

### 2.2 Model Development

#### 2.2.1 Model Structure Definitions

Three kinetic model structures were defined and evaluated using GRASP: simple, regulated, and detailed. Simple structures included the kinetic mechanism of each reaction considering only substrates and products (substrate-level regulation) observing reaction thermodynamics. Regulated structures incorporated additional allosteric regulations on top of the simple kinetic model structures. Finally, detailed structures considered the previous regulation but also accounted for kinetic mechanisms encompassing multiple reactions catalyzed by single enzymes, like the case of the promiscuous enzyme CrtYB. The differences between each structure are summarized in Table 1, while the summary of mechanisms for each reaction is presented in the Supporting Information Table S3. An illustration of the changes in the mechanisms used as input is presented in Figure 4.

**Table 1:** Regulatory Interactions and Enzyme Mechanisms Considered in the Evaluated Model Structures^a^

model structure				simple	regulated	detailed
substrate-level regulation						
reaction/gene	inhibitor molecules	inhibition type	reference			
ERG13	aacoa, coa	competitive	^53^	X	√	√
ERG12	grpp, frpp, ggpp	competitive	^54^	X	√	√

allosteric regulation						
reaction/gene	allosteric effector	effect	reference			
HMG2	Ggpp	negative	^55^	X	√	√

enzyme mechanisms					
reaction/gene	promiscuous reactions	reference			
ERG9	ERG9a, ERG9b	^56^	X	X	√
ERG20	ERG20a, ERG20b	^57^	X	X	√
CrtI	CrtIa, CrtIb	^58^	X	X	√
CrtYB	CrtBa, CrtBb, CrtYa, CrtYb	^59^	X	X	√

a Both enzyme mechanisms and regulation (inhibition) are implemented as catalytic information encoded in the reaction patterns of GRASP. Allosteric regulation is included as conformational information. Although HMG2 regulation consists of a protein degradation mechanism, it was modeled as an allosteric regulation to enable its inclusion within the GRASP framework.

**Figure 4:**
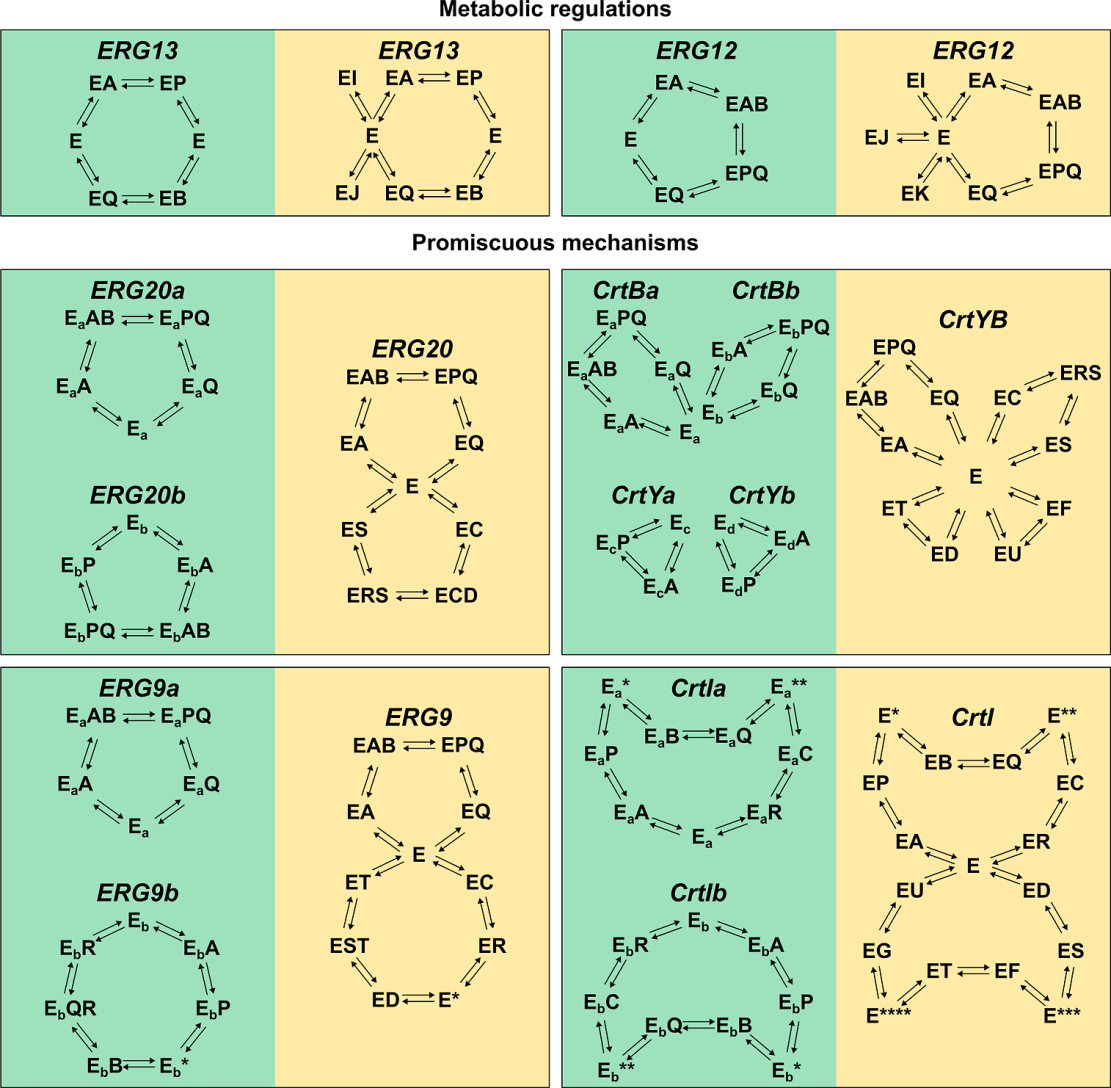
Reaction patterns used in the evaluated model structures. In green are depicted simple reaction patterns. Modifications including allosteric regulation and/or promiscuous mechanisms are shown in yellow. In all panels, E represents the free enzyme, whereas E* represents alternate free enzyme states, e.g., due to conformational changes. Subscripts underline that nonpromiscuous reaction mechanisms ignore the fact that the same enzyme performs multiple reactions. A, B, C, D, F and G represent reaction substrates, whereas P, Q, R, S, T and U denote reaction products. Finally, I, J and K, denote inhibitory metabolites.

#### 2.2.2 Simulation of Kinetic Model Ensembles

Kinetic model ensembles for every structure type were generated and simulated using the latest GRASP version^22^ as of July 30, 2024. Only minor modifications were added to the ABC rejection sampler.^21^ In short, a common reference condition of metabolic fluxes and metabolite concentrations was defined for each ensemble. Then, 10,000 model instances (also referred to as model particles) were sampled to generate the prior distribution of all the thermodynamically feasible rate constants and regulatory parameters.^23,25^ During sampling, the fluxes of new experimental conditions were calculated for every model particle generated. Briefly, relative enzyme and metabolite concentrations were computed within experimentally feasible bounds such that the kinetic model was able to comply with the steady state condition, i.e., no accumulation of balanced metabolites.^21^ Model particles that did not comply with this assumption were discarded. For the relative enzyme concentrations, the initial guessed value was set to the mean of the experimental relative transcript concentration, whereas its allowable range of variation was set to the experimentally determined 95% confidence interval. For the relative metabolite concentrations, the initial guessed value was assumed to be 1, i.e., equal to the reference condition, and the allowable range was defined as 0.1 to 10 for the unbalanced (i.e., external) metabolites, and 0.01 to 100 for balanced metabolites.

Finally, to simulate a sample from the posterior parameter distribution of each model structure, the most accurate 1% of the model particles (100 particles per ensemble) were selected based on their agreement with the experimental data.^60^ The latter cutoff was chosen for estimating the ABC-posterior during the rejection step based on previous choices for models of a similar size,^22,24^ and represented a reasonable trade-off between accuracy and computation time (see Computational implementation). Although this sample size is likely small to fully characterize the parameter space, it allows for a reasonable exploration of the system’s dynamics,^22–24,63^ which is the goal of this study. Indeed, despite the universally sloppy parameter sensitivities and poor identifiability of kinetic models,^61,62^ these features do not necessarily compromise the prediction power of ensemble models.^17,19^ To determine the most accurate models, a discrepancy measure between the observed and simulated fluxes in the new experimental conditions was calculated for every model particle. Instead of using a weighted infinite-norm as in Saa and Nielsen,^21^ here we used a weighted Euclidean norm as the discrepancy measure, which is already implemented in the latest version of GRASP.^22^

#### 2.2.3 Thermodynamic and Enzymatic Data Collection

The thermodynamic data of all the reactions in the metabolic pathway were estimated using the online tool eQuilibrator based on Component Contribution Method.^64^ The standard Gibbs’ free energies of reactions were calculated assuming an ionic strength of 0.2 M, a pMg of 3, and a pH of 6.8.^65^ The reaction mechanism, substrate-level, and allosteric regulations of each enzyme were obtained from the literature (refer to Supporting Information Table S3).

#### 2.2.4 Definition of Feasible Metabolite Concentration Ranges

Two types of metabolites were defined: unbalanced and balanced. Unbalanced (external) metabolites are compounds that participate in other metabolic reactions but are not present in the production pathway and therefore cannot be internally balanced. On the other hand, balanced metabolites are considered to participate only in the metabolic reactions within the pathway. Physiological concentrations for unbalanced metabolites were set to constrain the model to feasible values. Minimum and maximum concentration values were based on reported data in the literature (Supporting Information Text S6 and Table S19). Balanced metabolites were given an arbitrary range of 10^–9^ to 10^–1^ M for the prediction of new metabolic states to avoid over constraining the model except for lycopene. The lower bound of this metabolite was expanded to 10^–11^ to allow carotenoids fluxes to be thermodynamically feasible.

#### 2.2.5 Metabolic Control Analysis

The posterior distributions of the ensemble models were used to analyze the control structure at the reference condition. The control exerted by the enzymes of the pathway was estimated using flux and concentration response coefficients within the MCA framework.^66^ The coefficients of every model particle were calculated using GRASP functionalities.^22^ Due to the relatively small sample size of the control coefficients (100 model particles per ensemble), the distribution of the control coefficient means was estimated using bootstrapping with a resample size of 10^6^. Bootstrapping was used to evaluate the statistical significance of the calculated control coefficients means from the posterior ensemble^67^ and reduce the bias in their analysis due to the relatively small sample size. The statistical significance was evaluated using the percentile method along with the distributions obtained from the bootstrapping.^68^ Since statistical tests were executed for multiple variables associated with the same set of particles, Bonferroni correction was applied to avoid control Type I error (false positive) when conducting multiple comparisons.^67^

#### 2.2.6 Simulation and Evaluation of Production Scenarios

Relative enzyme and metabolite concentrations of selected ensembles were modified to simulate their impact on the production pathway. For enzyme perturbations, the process was analogous to the flux calculation under new experimental conditions during the generation of the ensembles. Each enzyme was perturbed one-at-a-time while the rest of the enzymes were restricted to a relative concentration value of nearly 1. To avoid overconstraining the model, the relative enzyme concentrations were allowed to vary 1% from the original set value. For metabolite perturbations, only one perturbation step was executed per simulation. The variation of the disturbed metabolite was constrained to 1% of the final relative concentration. The bounds of all enzymes were allowed to vary 10% of the set original to avoid over constraining the models.

### 2.3 Computational implementation

All the calculations and scripts required were implemented in the MATLAB 2021a environment.^69^ Flux Balance Analysis (FBA) was performed using the COBRA Toolbox v.3.0.^70^ The linprog LP solver included in MATLAB Optimization Toolbox was used for the estimation of the feasible Gibbs free energy ranges using Thermodynamics-based Metabolic Flux Analysis (TMFA).^25,71^ Simulations of the new experimental data and production scenarios were performed using the NLopt toolbox,^73^ particularly the SLSQP algorithm for constrained nonlinear optimization.^74^ Initial computations were executed on a Samsung Galaxy Book2 Pro (2.1 GHz Intel Core i7 12-Core, 16 GB RAM, Microsoft Windows 11, x86-based architecture). The ABC-rejection sampling and ensemble simulations were run on a node of the Unix-based Cluster of the School of Engineering at the Pontificia Universidad Católica de Chile (more details for the computational resources employed can be found in Supporting Information Text S7 and Table S20). Computational jobs were divided and distributed using the MATLAB Parallel Computing Toolbox mediated via SLURM.^72^ The execution time for each ensemble depended on its model structure. Simple model structures required 240 parameters, whereas regulated and detailed structures 252 parameters. These parameters encompassed microscopic kinetic constants, allosteric parameters, promiscuous enzyme activities, and Gibbs free energies of reaction (the latter are not known and must be sampled for simulating the system). For example, computing the posterior ensemble of the detailed models required evaluation times of up to 40 days using a 32-core cluster. Finally, the code and models generated here can be found and downloaded for free from the GitHub repository https://github.com/SysBioengLab/BcarGRASP.

## 3 Results and Discussion

### 3.1 Phenotypic Characterization of Recombinant Yeast Strains for Kinetic Modeling

Experimentally determined fluxes for the three β-car strains at the two growth rates are shown in Table 2. The rates of production and consumption of extracellular metabolites (e.g., glucose, ethanol, glycerol, and acetate) can be found in Supporting Information Table S4. As expected from the results of López,^28^ β-car4 outperformed the other strains in carotenoid production at the two growth rates. In all cases, the accumulation of β-carotene was favored over lycopene at a high dilution rate. The opposite occurred at a low dilution rate, except for β-car2.

**Table 2:** Experimental Growth and Carotenoid Production Rates of Different Recombinant Yeast Strains at Different Dilution Rates

				production rate (nmol/gDCW/h)^b^		
growth rate (1/h)^a^			lycopene		β-carotene	
Strain	mean	95% CI^c^	mean	95% CI	mean	95% CI^c^
β-car2	0.101	[0.0971, 0.1048]	94.4	[85.4, 104.4]	151.3	[140.3, 163.4]
	0.254	[0.2507, 0.2580]	84.3	[74.1, 94.9]	184.2	[138.8, 230.5]
β-car3	0.101	[0.0971, 0.1048]	472.0	[430.7, 518.5]	227.1	[207.6, 240.9]
	0.254	[0.2507, 0.2580]	126.5	[123.2, 129.9]	294.8	[198.6, 391.4]
β-car4	0.101	[0.0971, 0.1048]	760.7	[647.5, 877.1]	185.3	[166.4, 201.8]
	0.254	[0.2507, 0.2580]	392.9	[352.6, 434.6]	453.6	[361.2, 547.4]

a Mean dilution rates were used as the observed specific growth rate under different conditions.

b The mean experimental production rates of lycopene and β-carotene along with the specific growth rate was employed for the calculations of the pathway flux.

c CI denotes the 95% confidence interval of each measured rate.

The experimental conditions of the pathway were divided by growth rate. In each case, the β-car4 strain data was used as reference condition for the model due to its larger carotenoid yield. β-car3 and β-car2 were utilized as additional experimental conditions. Consequently, the transcriptomic profiles were normalized against the expression of β-car4 at each growth rate. The heatmaps of relative mRNA concentration are shown in Figure 5. Supporting Information Tables S5 and S6 include the confidence intervals of the relative mRNA abundances. The mRNA quantified for the heterologous genes was consistent with the number of copies inserted in the genome (Figure 3). At low growth rate, the average relative expressions of CrtE, CrtI and CrtYB were respectively ∼61%, ∼89% and ∼68% in β-car3. In contrast, the estimated expression values for the latter set of genes were ∼27%, ∼51% and ∼33% for β-car2.

**Figure 5:**
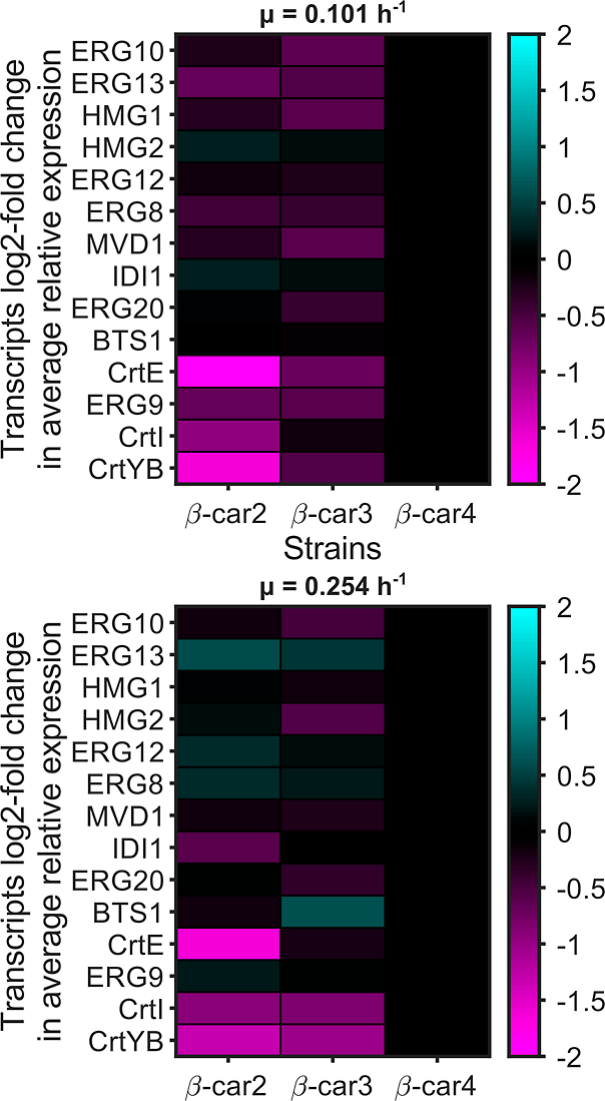
Mean relative gene expression of the production pathway for different recombinant yeast strain at different growth rates. Heatmaps depict the gene expression relative to the β-car4 expression at 0.101 h^–1^ (top) and 0.254 h^–1^ (bottom) growth rates. Overall, the mRNA quantified for the heterologous genes was consistent with the number of copies integrated into the genome of each strain.

### 3.2 Evaluation of Kinetic Model Ensembles

Six ensemble models were generated using the ABC-GRASP framework and rejection sampler.^75^ Thermodynamically feasible kinetic models^25,76^ anchored at the β-car4 strain at either low or high dilution rates were simulated. Different model structures (simple, regulated, and detailed) were used in each scenario. The relative transcriptomic, metabolic and flux data of the β-car3 and β-car2 strains at the corresponding dilution rate conditions were used to simulate and fit the models under new experimental conditions, enabling selection of more accurate model instances based on their discrepancy scores (Supporting Information Figure S1). To evaluate the performance of posterior ensembles, a cross-validation methodology was implemented. For this task, only one of the β-car3 and β-car2 strains was used for model training during the rejection process and the remaining was used for validation (Supporting Information Figure S2). In all cases, the posterior discrepancy scores distribution had a lower median than the particles from the prior distribution (*p*-value < 0.01, Rank Sum test, Supporting Information Figure S2), which supported the application of the rejection step.

Analysis of the posterior ensembles revealed that the detailed kinetic structures had the lowest discrepancy score under both growth conditions (Supporting Information Figure S1). The discrepancy scores ranged from 0.239 to 0.331 and 0.228 to 0.298 for the detailed ensembles under the low and high dilution rates, respectively. Notably, the ensembles with detailed kinetics generated model particles that simulated the experimental fluxes within their 95% confidence intervals (Figure 6a), except for SK_lyc at high growth rate (Figure 6b). In contrast, the simple and regulated models were unable to predict the SK_b_car and SK_lyc fluxes at low growth rate (Figure 6a) and the ERG9b flux at high growth rate (Figure 6b). In fact, the latter ensembles lacked the ability to properly simulate the strains under the different growth rates. Including more complex kinetics in enzyme-catalyzed reactions (e.g., enzyme promiscuity) enables a broader exploration of potential metabolic states (see Supporting Information, Figure S3). However, this additional flexibility comes at the cost of reduced computational efficiency (refer to C. implementation). Here, such broad exploration is required for finding kinetic parametrizations that explain the observed experimental fluxes (Figure 6a).

**Figure 6:**
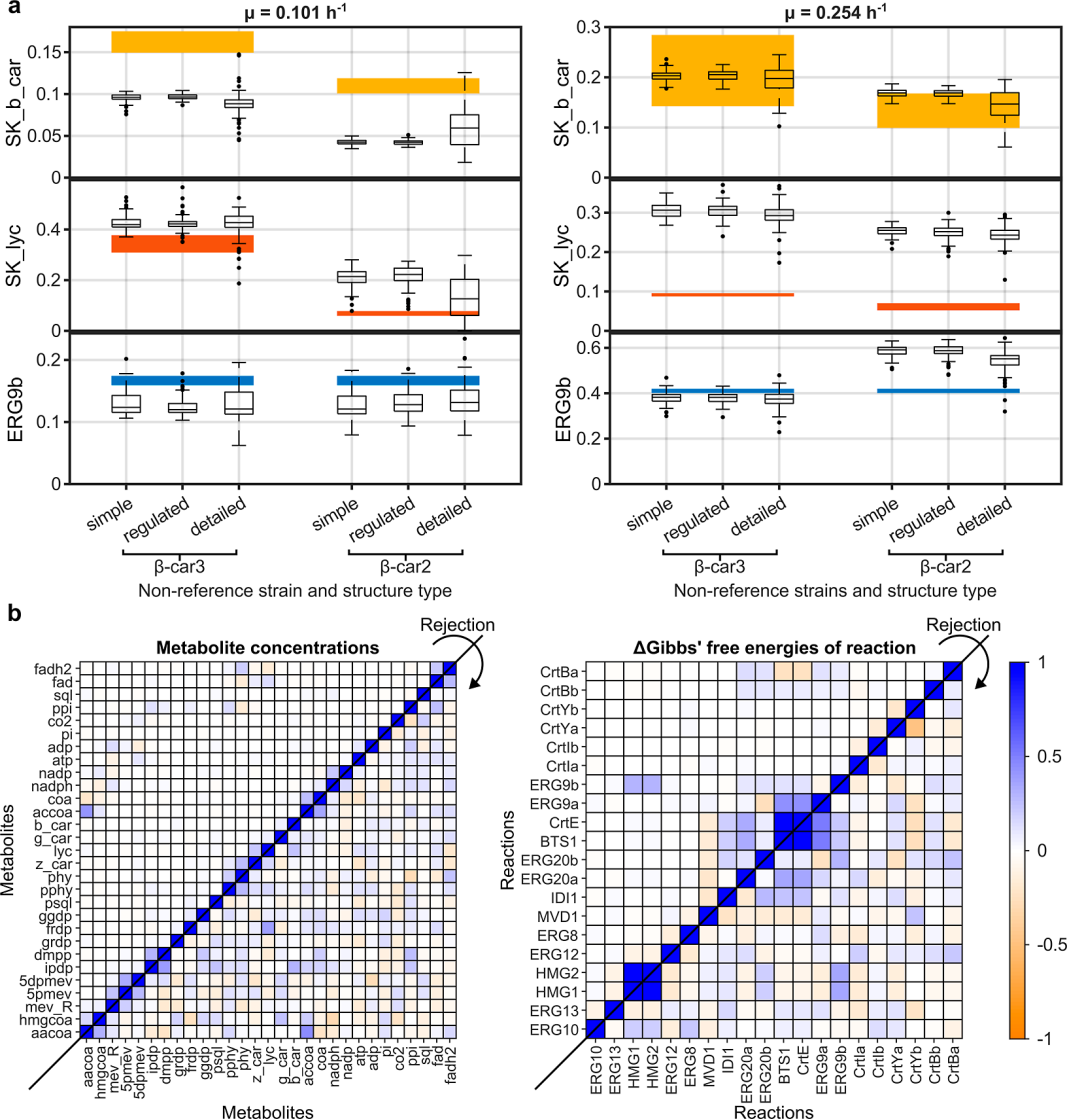
Prediction of kinetic model ensembles trained with ABC-GRASP using the rejection sampler. (a) Boxplots of the free fluxes of the modeled pathway (SK_b_car, SK_lyc and ERG9b) simulated with the different posteriors described by the kinetic ensembles in different experimental conditions, namely: β-car 3 and β-car2. The reference condition (β-car4) is excluded in the two scenarios as all ensembles are anchored at this point by construction. The models sampled and selected for low and high growth rates are presented on the left- and right-hand sides, respectively. All fluxes are represented in mmol/L/h. Solid colored rectangles represent the 95% confidence interval of the experimentally measured fluxes for each strain. (b) Heatmaps summarizing the change in the spearman rank correlation between metabolite distributions (left) and Gibbs free energy of reactions (right) obtained from the detailed models before and after the execution of the rejection sampler. Only the models sampled and selected for low growth are depicted. In each heatmap, the correlations of the prior ensembles are included in the upper left-hand corner, while the correlations of the posterior ensembles are presented in the lower right-hand corner. An enrichment in the correlation structure of the pathway metabolites can be observed as new experimental data is integrated.

Further analysis of the posterior ensembles revealed that the rejection process did not substantially affect the metabolite concentration and Gibbs free energies of reaction (marginal) distributions nor their ranges (Supporting Information Figures S4 and S5). Similar results were encountered overall for the relative values of metabolite concentrations and enzyme abundances in the nonreference conditions (Supporting Information Figures S6 and S7). However, the rejection did select and cause a general change in the correlation structure of the metabolite concentrations and Gibbs free energy of reactions, specifically an increase in the average absolute correlations at low (Figure 6b) and high dilution rates (Supporting Information Figure S8). This suggests that parameter inference primarily promoted learning of higher-order relationships between parameters and states, rather than remodeling the prior marginals, i.e., means. In this case, the rejection process seemingly enables to establish coupling relationships that are required for the network to consistently operate at steady state and to explain the observed data. A clear example of the latter is the enrichment in the concentration correlations of accoa and aacoa and the rest of the network metabolites (Figure 6b and Supporting Information Figure S8). While these metabolites in the prior ensembles only display correlations with each other, the metabolites in the posterior ensembles display noticeable correlations with most pathway metabolites. A similar pattern is observed for the Gibbs free energy differences of BTS1 and CrtE (Figure 6b and Supporting Information Figure S8). The learning of higher-order relationships is indeed consistent with recent findings that indicate a strong agreement between the latter statistics for prior samples in GRASP and reported kinetic information for single enzymes.^23^ On the other hand, this also suggests that building an accurate model capable of explaining multiple data sets may be more difficult to achieve and may be insufficiently informed by reference state data alone.

### 3.3 Revelation of Flux Limitations in β-Carotene Production

MCA was executed to identify enzymes that exert high control over β-carotene accumulation in *S. cerevisiae*. For the latter task, the detailed kinetic ensembles were chosen because of their ability to account for enzyme promiscuity and to more accurately describe production fluxes. The averages of the flux response coefficients and concentration response coefficients were estimated for the strain β-car4 (the highest β-carotene producer) at low and high growth rates (Figure 7a, refer to Supporting Information Figure S9 for the descriptions of enzymes, fluxes, and metabolites). High control coefficients associated with lycopene production were also analyzed due to its cytotoxic effect that may impair cellular growth.^77^ Thus, enzyme perturbations that both diminish lycopene and boost β-carotene production were prioritized (Figure 7b). Flux and concentration response coefficients resulted in consistent values for these two compounds, as both are directly linked through reaction sinks used to describe their accumulation in the cell. For both dilution rates, MCA indicated that ERG13 and CrtYB had statistically significant effects on lycopene and β-carotene production (Figure 7c). ERG13 has a positive effect on lycopene, while it has a negative influence on β-carotene. The opposite was observed for CrtYB.

**Figure 7:**
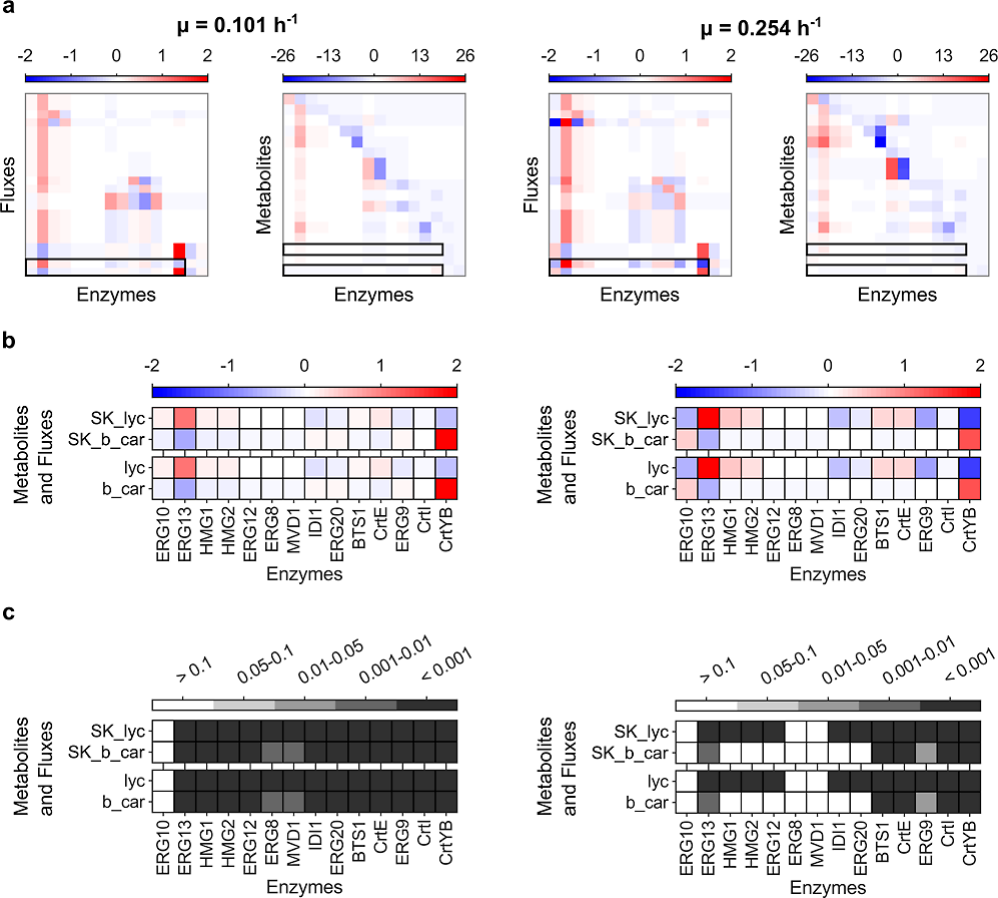
Metabolic control analysis of the detailed posterior kinetic model ensemble. The analysis of ensembles for low and high growth rates are presented on the left- and right-hand side, respectively. (a) Average flux and concentration control coefficients of the metabolic components of the pathway. The black rectangles enclose the coefficients of interest for β-carotene synthesis. Supporting Information Figure S9 includes the names of enzymes, fluxes, and metabolites. (b) Heatmaps of average control coefficients related to the fluxes and concentrations of β-carotene and lycopene. (c) Statistical significance of the estimated control coefficients. Heatmaps depict *p*-values estimated using the bootstrapping percentile method with Bonferroni correction.

The posterior kinetic ensembles predict that ERG10 does not exert substantial control over the system, even though it is the entry point to the pathway. In contrast, ERG13 is predicted to exert higher control. Notably, the ensemble includes the substrate-level inhibition by aacoa and coa in the reaction mechanism of ERG13 that prevents accoa binding, which may help to explain its relative control.^53,78^ Indeed, higher expression of ERG10 might cause aacoa accumulation due to the competitive inhibition at ERG13.^53^ This is consistent with ERG10 being described as already constitutively expressed, as opposed to the weaker expression of ERG13.^79^ Accordingly, the results suggest that ERG13 is acting as the controlling enzyme of the overall flux entering the pathway,^80^ and that this flux will be directed primarily toward lycopene production (Figure 7b). Meanwhile, CrtYB is a bottleneck downstream of lycopene in the β-car4 strain, and increasing its expression is necessary for increasing β-carotene production (Figure 7b).

### 3.4 Evaluation of Metabolic Scenarios for Higher β-Carotene Production

To evaluate the effect of larger perturbations in the system, the detailed kinetic model ensembles were used to simulate perturbations of the relative enzyme abundances and metabolite concentrations (Figure 8a). ERG13 and CrtYB included additional perturbations due to the higher predicted control exerted on the system. The same was applied to accoa provided that is a common target for enhancing precursor supply and increasing pathway flux.^81^ Most of the simulated step changes were defined so that the perturbations were similarly spaced between each other on a log2 scale. The exception being the Crt genes, whose relative modifications were based on the number of gene copies present in β-car4.

**Figure 8:**
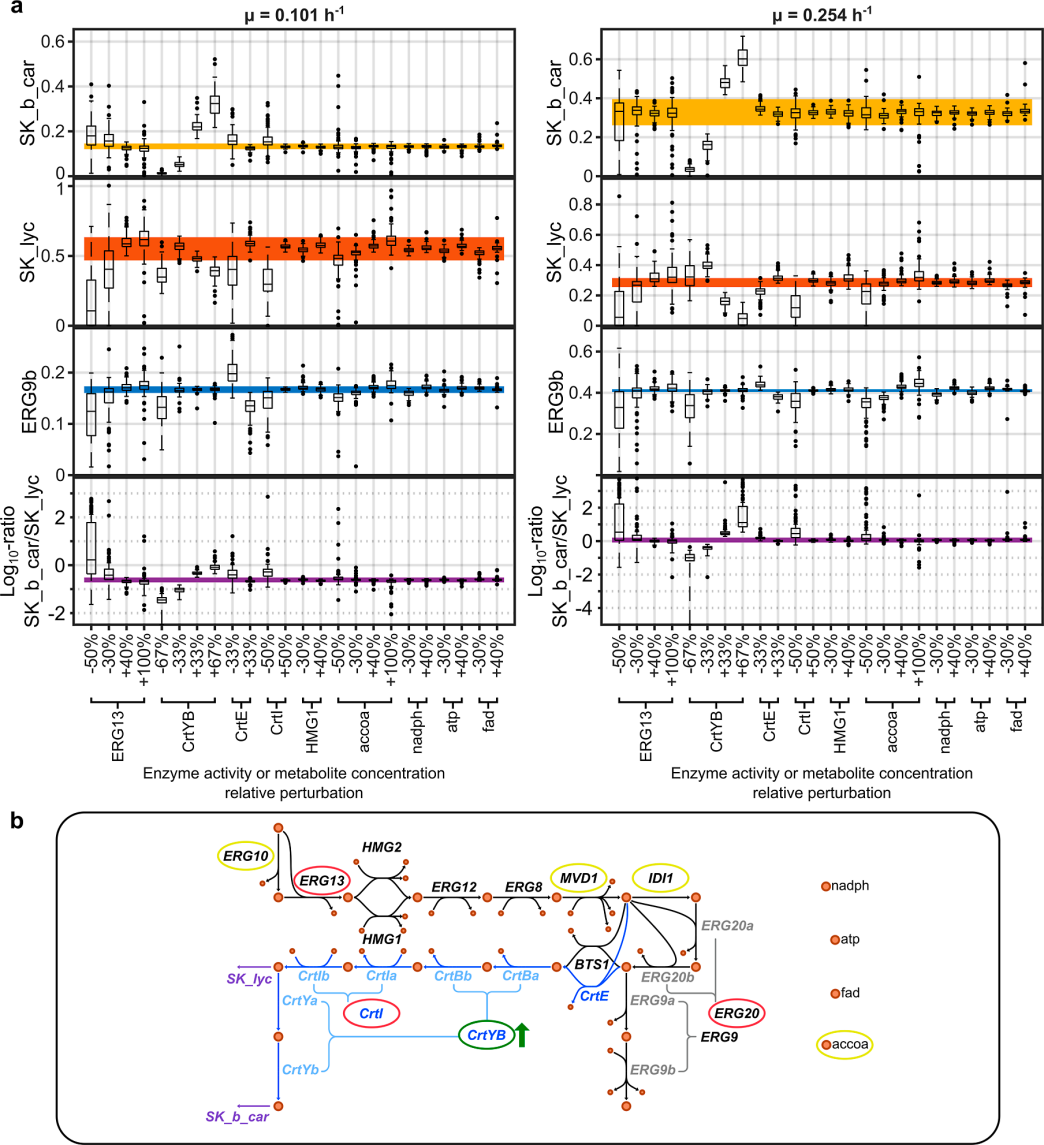
Simulations of different perturbations using the detailed posterior kinetic model ensembles. (a) Boxplots of the simulated fluxes SK_b_car, SK_lyc, and ERG9b for several perturbations. The results for low and high growth rates are presented on the left- and right-hand sides, respectively. The log10 ratio between SK_b_car and SK_lyc is also included to emphasize changes in the proportion of β-carotene and lycopene production. Solid colored rectangles represent 95% confidence intervals of the experimentally measured fluxes for the reference strain β-car4 (highest producer). All fluxes are represented in mmol/L/h. Only the discussed simulations are included in the figure. Supporting Information Figures S10 and S11 include the complete set of simulations. (b) Components of the network that exert metabolic control over β-carotene synthesis. The most promising target for increasing β-carotene production (CrtYB) is circled in green. Enzymes and metabolites that were calculated to have medium potential to limit production are circled in yellow. Enzymes that were classified as having high potential of constraining production are circled in red.

ERG13 decrease did not have a consistent impact at different growth rates across the ensembles. Although there are some model particles that predict higher β-carotene and lower lycopene production, the opposite is also observed. While this is partially consistent with the MCA results, it highlights the limitation of the latter analysis beyond the vicinity of the studied refence state. Indeed, the control structure of a metabolic pathway is a local property of the system that varies with changes in the expression of flux-controlling enzymes. Most of the simulations indicate that the ERG9b flux, which leads to sterol biosynthesis,^79^ would be negatively affected when downregulating ERG13. This can compromise ergosterol production, which is an essential component of the cell membrane.^43^ Its depletion can impair growth and be detrimental to the overall carotenoid production in bioreactor cultivations.^81^ Overall, the simulations suggest that ERG13 is not a convincing target for enhancing β-carotene production in the β-car4 strain (reference strain).

According to the simulations, CrtYB is the most promising candidate target for increasing β-carotene production. Notably, at both dilution rates, the ensemble consistently predicts an increase in SK_b_car for a positive step change, i.e., enzyme upregulation. All the simulations indicated that the predicted flux will be above the 95% confidence interval of the reference condition. A 67% upregulation in CrtYB activity led to ∼145.6% and ∼84.2% relative increases in SK_b_car at low and high growth rates, respectively. Moreover, the mean log10 ratio between SK_b_car and SK_lyc trended upward from −0.617 to −0.072 for the model ensemble at 0.101 h^–1^ growth rate and climbed from 0.06 to 1.49 for the model ensemble at 0.254 h^–1^ growth rate. In contrast, the ERG9b flux does not seem to be overly influenced by these perturbations. The ERG10 flux also presented little variation in the ensembles (Supporting Information Figure S10), implying that the CrtYB perturbation does not cause a large change in the overall pathway flux. Consequently, the predicted increase in β-carotene production corresponds to a redirection of flux from lycopene accumulation, suggesting an increase in the selectivity of the system. Additionally, this perturbation is not expected to have a substantial impact on the flux toward ergosterol synthesis. Altogether, the results suggest that more copies of the CrtYB gene would be beneficial to improve β-carotene production in the reference strain. In fact, the activity of this enzyme has been previously identified as a limiting factor in carotenoid synthesis,^82–84^ and the analysis of the simulations is consistent with these findings. However, it is important to emphasize that the flux-controlling components identified by the models are specific to the metabolic conditions studied here. Thus, they may exert different control in other β-carotene-producing strains with different genetic backgrounds.

In the case of accoa, simulations of changes in its concentration showed slight variations in the synthesis of lycopene, ERG9b and ERG10 fluxes (Supporting Information Figure S11), but no major impact was observed in β-carotene production. The scale of the accoa step modifications is within the magnitude of interventions that alter the supply of this precursor,^85^ yet the lack of influence on β-carotene accumulation is likely due to the greater control exerted by CrtYB. Perturbation of cofactor concentrations, which are typically targeted in metabolic engineering,^86^ did not significantly affect the pathway flux in this system (Supporting Information Figure S11).

The upregulation of CrtI and CrtE in the simulations had no relevant effect on the pathway. However, their downregulation mostly predicted an increase in β-carotene and a decrease in lycopene production at a low growth rate. This is an example of how simultaneous overexpression of heterologous enzyme genes in an almost linear pathway may not lead to higher production, and conversely, balanced expression should be achieved to avoid potentially detrimental accumulation of metabolic intermediates.^87^

### 3.5 Response Simulations to Increased Metabolic Perturbations

The tested perturbations of the model point to a lack of influence of HMG1 over β-carotene synthesis. This contrasts with previous literature, which indicates that overexpression of tHMG1 (a truncated version of the enzyme) usually improves carotenoid production.^28,81,88^ Indeed, there are known cases where this does not apply.^89^ This difference could be explained due to the nature of the HMG1 regulatory mechanism, which cannot be properly captured by the regulatory mechanisms supported by GRASP.^22^ This enzyme is regulated at the translational level by a negative feedback system that depends on the 5′ untranslated region of its mRNA,^90^ which is certainly not associated with the enzymatic activity itself. Furthermore, tHMG1 is typically expressed under the influence of a strong constitutive promoter.^28,88,91,92^ The combined constitutive expression and absence of translational regulation may well contribute to tHMG1 having an overall enzyme activity that is at least an order of magnitude larger than the native HMG1.^91,93^ Besides, tHMG1 lacks HMG1 binding site to the endoplasmic reticulum membrane,^94^ which causes further differences in the functioning of both enzymes. To test if the detailed kinetic model ensembles could emulate the impact of the inclusion of tHMG1 in the network, simulations were carried out that increased the activity of the enzyme HMG1 from 10-fold up to 100-fold the value of the reference state (Supporting Information Figure S12). For both low and dilution rates, there were overall no consistent responses in the flux toward β-carotene or lycopene synthesis. However, there were model particles that predicted an increase of up 2-fold the reference flux toward β-carotene production (Supporting Information Figure S12). The simulated increases in HMG1 are comparable to the expression of tHMG1,^91,93^ and thus, offered additional support for the detailed kinetic model structures.

Simulation of a 67% CrtYB upregulation (hereafter referred to as Base CrtYB state), which roughly translates to the addition of two copies of this gene, did not cause substantial changes in other variables. Neither relative unbalanced metabolite concentrations nor other enzyme abundances showed major adjustments compared to the reference state (Supporting Information Figure S13). To determine if there are factors that could become limiting to β-carotene generation in the Base CrtYB state, a series of new simulations were performed (Supporting Information Figure S14 for enzyme abundance perturbations, and Supporting Information Figure S15 for metabolite concentration perturbations). Each simulation included the 67% CrtYB upregulation with an additional metabolic change like the modifications presented in Figure 8a. Notably, no other perturbation achieved a significant increase in the SK_b_car flux, suggesting that even in the Base CrtYB state, the latter enzyme remains as the main flux control checkpoint. Interestingly, some model simulations indicated possible negative effects toward β-carotene synthesis.

To identify possible performance threats for the Base CrtYB state, a robustness criterion was defined using the confidence intervals of the reference state as threshold. If more than 5% of the kinetic models in the ensemble led to a SK_b_car flux lower than the threshold, the enzyme or metabolite was deemed as having medium potential for becoming limiting. On the other hand, if more than 20% of the models met the above criterion, the component was deemed to have high potential to become limiting. ERG10, MVD1, IDI1, and accoa were recognized as having medium potential, while ERG13, CrtI, and ERG20 had high potential (Figure 8b). The effect of CrtI differed from the initial simulation results. Results suggest that a decrease in CrtI is favorable for redirecting flux from lycopene in the reference strain, but in the Base CrtYB scenario, deleting a copy of CrtI is predicted to limit the flux toward β-carotene. This fact again emphasizes that strain design should consider the genetic and metabolic backgrounds of the producer strain to implement effective and optimal metabolic interventions.

## 4 Conclusions and Outlook

Kinetic models are powerful tools for rationally understanding complex metabolic systems and informing strain designs. Simulation-based approaches such as ABC-GRASP enable exploration of the thermodynamically feasible flux space spanned by detailed kinetic models, while appropriately capturing parameter and prediction uncertainty when informed with multiomics datasets. Parameter inference within the ABC framework enables selection of more accurate models that explain new metabolic states using different model structures, providing mechanistic insights about the system. Based on these capabilities, here we evaluated different kinetic model structures describing β-carotene production by recombinant *S. cerevisiae* strains to identify flux bottlenecks as well as promising metabolic interventions for enhanced accumulation. Our results supported that detailed kinetic models including both allosteric regulation and complex mechanistic descriptions (e.g., enzyme promiscuity) are necessary to explain the metabolic phenotype of different production strains in different conditions. Metabolic control analysis of the detailed models revealed that the promiscuous CrtYB enzyme exerts the highest control over β-carotene production at different growth conditions in the high producer reference strain (β-car4). Simulation of various enzyme and metabolite perturbations confirmed these results and discarded other seemingly intuitive targets for intervention, e.g., upregulation of ERG10—the entry point to the mevalonate pathway. Importantly, model simulations emulating the inclusion of tHMG1 in the pathway showed an increased flux toward β-carotene in some instances, which is consistent with experimental observations in strains with similar genetic background. A more consistent intervention for higher β-carotene production was the 67% upregulation of CrtYB—which is equivalent to the addition of two additional copies of this gene. This strategy showed great promise for improving β-carotene production without a significant negative effect on sterol synthesis. Interestingly, this strategy did not work well when paired with the counterintuitive downregulation of CrtI—which alone showed a slightly positive effect on β-carotene production. These results highlight the need for taking into consideration the genetic and metabolic backgrounds of producer strains before metabolic intervention. For this task, computational frameworks such as ABC-GRASP are excellent tools for *in silico* metabolic prospection and rational design of genetic interventions.

## Supplementary Material



## Data Availability

Data and code availability The codes for preprocessing, data generation for figures and tables, and model building using GRASP are freely available at https://github.com/SysBioengLab/BcarGRASP.

## Notes

Additional experimental results are presented in Supplementary Tables, Figures, and Texts. Supplementary Tables include the abbreviations of the model components, as well as the fluxes and relative transcripts abundances used for sampling. Supplementary Figures include rejection results, model cross-validation, flux comparisons of the evaluated models, priors and posteriors prediction comparisons, metabolic control coefficients, and flux simulations of different perturbations around the reference states. Supporting Information describes methods for the estimation of dry cell weight, chemostat operation, transcripts quantification, metabolic rates calculation, and relative enzyme abundances assessment (PDF)
