# Supplementary material for: Complex Kinetic Models Predict β‑Carotene Production and Reveal Flux Limitations in Recombinant Saccharomyces cerevisiae Strains
Source: ACS Synth Biol. 2025 Sep 2;14(9):3457–72. doi: 10.1021/acssynbio.5c00256 (PMC12455641; doi:10.1021/acssynbio.5c00256)
Supplement: Supplementary file 1 [file sb5c00256_si_001.pdf]

Supporting Information

Complex kinetic models predict β-carotene production and reveal flux limitations in recombinant *Saccharomyces cerevisiae* strains

Benjamín R. Elizondo and Pedro A. Saa

[a]Departamento de Ingeniería Química y Bioprocesos, Escuela de Ingeniería, Pontificia Universidad Católica de Chile, 7820436, Santiago, Chile

[b]Instituto de Ingeniería Matemática y Computacional, Pontificia Universidad Católica de Chile, 7820436, Santiago, Chile

[*]Corresponding Author: Departamento de Ingeniería Química y Bioprocesos, Escuela de Ingeniería, Pontificia Universidad Católica de Chile, 7820436, Santiago, Chile.

Email: pnsaa@uc.cl

I. Supplementary Tables

Table S1. List of metabolite abbreviations.

Metabolite ID	Metabolite Name	Balanced
aacoa	Acetoacetyl-CoA	YES
hmgcoa	3-Hydroxy-3-methylglutaryl-CoA	YES
mev_R	Mevalonate	YES
5pmev	5-Phosphomevalonate	YES
5dpmev	5-Pyrophosphomevalonate	YES
ipdp	Isopentenyl pyrophosphate	YES
dmpp	Dimethylallyl pyrophosphate	YES
grdp	Geranyl pyrophosphate	YES
frdp	Farnesyl pyrophosphate	YES
ggdp	Geranylgeranyl pyrophosphate	YES
psql	Presqualene pyrophosphate	YES
pphy	Prephytoene pyrophosphate	YES
phy	Phytoene	YES
z_car	zeta-Carotene	YES
lyc	Lycopene	YES
g_car	gamma-Carotene	YES
b_car	beta-Carotene	YES
accoa	Acetyl-CoA	NO
coa	CoA	NO
nadph	NADPH	NO
nadp	NADP+	NO
atp	ATP	NO
adp	ADP	NO
pi	Orthophosphate	NO
co2	CO2	NO
ppi	Diphosphate	NO
sql	Squalene	NO
fad	FAD	NO
fadh2	FADH2	NO

Table S2. List of reaction and enzyme abbreviations. Substrates and products of each reaction are also included.

Enzyme ID	Reaction ID	Reaction name	Substrates	Products
ERG10	ERG10	acetyl-CoA C-acetyltransferase	2 accoa	aacoa + coa
ERG13	ERG13	hydroxymethylglutaryl-CoA synthase	accoa + aacoa	hmgcoa coa
HMG1	HMG1	hydroxymethylglutaryl-CoA reductase	hmgcoa + 2 nadph	R-mev + coa + 2 nadp
HMG2	HMG2	hydroxymethylglutaryl-CoA reductase	hmgcoa + 2 nadph	R-mev + coa + 2 nadp
ERG12	ERG12	mevalonate kinase	R-mev + atp	5pmev + adp
ERG8	ERG8	phosphomevalonate kinase	5pmev + atp	5dpmev + adp
MVD1	MVD1	pyrophosphomevalonate decarboxylase	5dpmev + atp	ipdp + adp + pi + co2
IDI1	IDI1	isopentenyl-pyrophosphate delta-isomerase	ipdp	dmpp
ERG20	ERG20a	farnesyl pyrophosphate synthase step I	dmpp + ipdp	grdp + ppi
ERG20	ERG20b	farnesyl pyrophosphate synthase step II	grdp + ipdp	frdp + ppi
BTS1	BTS1	geranylgeranyl pyrophosphate synthase	frdp + ipdp	ggdp + ppi
CrtE	CrtE	geranylgeranyl pyrophosphate synthase	frdp + ipdp	ggdp + ppi
ERG9	ERG9a	farnesyl-pyrophosphate farnesyltransferase	frdp + frdp	psql + ppi
ERG9	ERG9b	squalene synthase	psql + nadph	sql + nadp + ppi
CrtI	CrtIa	phytoene desaturase step I	phy + fad + fad	z_car + fadh2 + fadh2
CrtI	CrtIb	phytoene desaturase step II	z_car + fad + fad	lyc + fadh2 + fadh2
CrtYB	CrtBa	15-cis-phytoene synthase step I	ggdp + ggdp	pphy + ppi
CrtYB	CrtBb	15-cis-phytoene synthase step II	pphy	phy + ppi
CrtYB	CrtYa	lycopene beta-cyclase step I	lyc	g_car
CrtYB	CrtYb	lycopene beta-cyclase step II	g_car	b_car
SK_lyc	SK_lyc	lycopene sink	lyc	
SK_b_car	SK_b_car	beta-carotene sink	b_car	

Table S3. Reaction mechanisms and sources.

Reaction ID	Type	Sources	Comments
ERG10	Ping Pong Bi Bi	[1]	
ERG13	Ping Pong Bi Bi	[2]	
HMG1	Special	[3]	Similar to two consecutive Ordered Bi Bi
HMG2	Special	[3]	Similar to two consecutive Ordered Bi Bi
ERG12	Ordered Bi Bi	[4]	
ERG8	Random Bi Bi	[5]	
MVD1	Ordered Bi Quater	[6]	
IDI1	Ordered Uni Uni	[7]	
ERG20a	Ordered Bi Bi	[8]	
ERG20b	Ordered Bi Bi	[8]	
BTS1	Ordered Bi Bi	[9]	
CrtE	Ordered Bi Bi	[9]	
ERG9a	Ordered Bi Bi	[10]	
ERG9b	Special	[10]	Similar to Ping Pong Bi Bi with additional release
CrtIa	Ping Pong Ter Ter	[11]	Simplified from source, and assumed as Ping Pong
CrtIb	Ping Pong Ter Ter	[11]	Simplified from source, and assumed as Ping Pong
CrtBa	Ordered Bi Bi	[12]	
CrtBb	Ordered Uni Bi	[12]	
CrtYa	Ordered Uni Uni	[12]	
CrtYb	Ordered Uni Uni	[12]	

37

Table S4. Extracellular experimental fluxes. Mean measured fluxes for each strain and growth rate in chemostat cultivations. T
95% confidence interval of the fluxes is also included.

	Growth Rate (1/h)	Extracellular Fluxes (mmol/gDCWh)							
		Glucose		Ethanol		Acetate		Glycerol	
Strain	Mean	Mean	95% CI	Mean	95% CI	Mean	95% CI	Mean	95% CI
β-car2	0.101	-6.68	[-7.223, -6.176]	6.40	[5.873, 6.961]	0.639	[0.582, 0.700]	0.453	[0.424, 0.484]
	0.254	-14.40	[-15.54, -13.32]	16.12	[15.21, 17.21]	0.000	[0.000, 0.000]	1.353	[1.186, 1.526]
β-car3	0.101	-7.13	[-7.694, -6.580]	6.01	[5.725, 6.300]	0.623	[0.550, 0.698]	0.592	[0.528, 0.658]
	0.254	-11.72	[-17.79, -5.66]	15.84	[15.06, 16.64]	0.000	[0.000, 0.000]	0.000	[0.000, 0.000]
β-car4	0.101	-5.54	[-5.949, -5.157]	2.90	[2.697, 3.122]	0.518	[0.342, 0.697]	0.573	[0.512, 0.636]
	0.254	-14.28	[-15.69, -12.92]	14.55	[11.72, 17.68]	0.000	[0.000, 0.000]	0.000	[0.000, 0.000]

Table S5. Relative transcript abundances in chemostats at 0.101 h^{-1} dilution rate. Strains β-car2 and β-car3 are normalized to the reference strain β-car4 at the same dilution rate. Averages and their 95% confidence interval are included.

	**β-car2**			**β-car3**		
Gene ID	Lower 95% CI	Mean	Upper 95% CI	Lower 95% CI	Mean	Upper 95% CI
ERG10	0.800	0.850	0.904	0.522	0.638	0.779
ERG13	0.597	0.625	0.653	0.476	0.686	0.988
HMG1	0.669	0.815	0.992	0.500	0.660	0.872
HMG2	0.975	1.213	1.510	0.927	1.108	1.325
ERG12	0.651	0.892	1.222	0.582	0.855	1.256
ERG8	0.543	0.744	1.019	0.574	0.769	1.031
MVD1	0.693	0.827	0.986	0.575	0.664	0.766
IDI1	0.915	1.197	1.567	1.006	1.121	1.248
ERG20	0.929	1.056	1.200	0.663	0.762	0.876
BTS1	0.893	0.966	1.045	0.800	0.937	1.097
CrtE	0.203	0.271	0.362	0.431	0.615	0.876
ERG9	0.593	0.633	0.675	0.459	0.661	0.952
CrtI	0.422	0.511	0.618	0.680	0.891	1.167
CrtYB	0.308	0.328	0.349	0.625	0.685	0.751

Table S6. Relative transcript abundances in chemostats at 0.254 h^{-1} dilution rate. Strains β-car2 and β-car3 are normalized to the reference strain β-car4 at the same dilution rate. Averages and their 95% confidence interval are included.

	**β-car2**			**β-car3**		
Gene	Lower 95% CI	Mean	Upper 95% CI	Lower 95% CI	Mean	Upper 95% CI
ERG10	0.509	0.889	1.553	0.459	0.723	1.138
ERG13	0.452	1.490	4.908	0.225	1.342	8.003
HMG1	0.439	1.047	2.496	0.318	0.887	2.477
HMG2	0.558	1.125	2.266	0.359	0.677	1.279
ERG12	0.409	1.283	4.021	0.235	1.113	5.262
ERG8	0.431	1.270	3.744	0.293	1.190	4.835
MVD1	0.690	0.900	1.176	0.591	0.848	1.216
IDI1	0.157	0.660	2.780	0.198	0.992	4.967
ERG20	0.704	1.027	1.497	0.471	0.777	1.282
BTS1	0.517	0.896	1.554	0.829	1.559	2.933
CrtE	0.213	0.323	0.489	0.352	0.875	2.176
ERG9	0.301	1.191	4.711	0.197	1.035	5.434
CrtI	0.319	0.529	0.878	0.212	0.561	1.484
CrtYB	0.147	0.399	1.087	0.135	0.483	1.737

II. Supplementary Figures

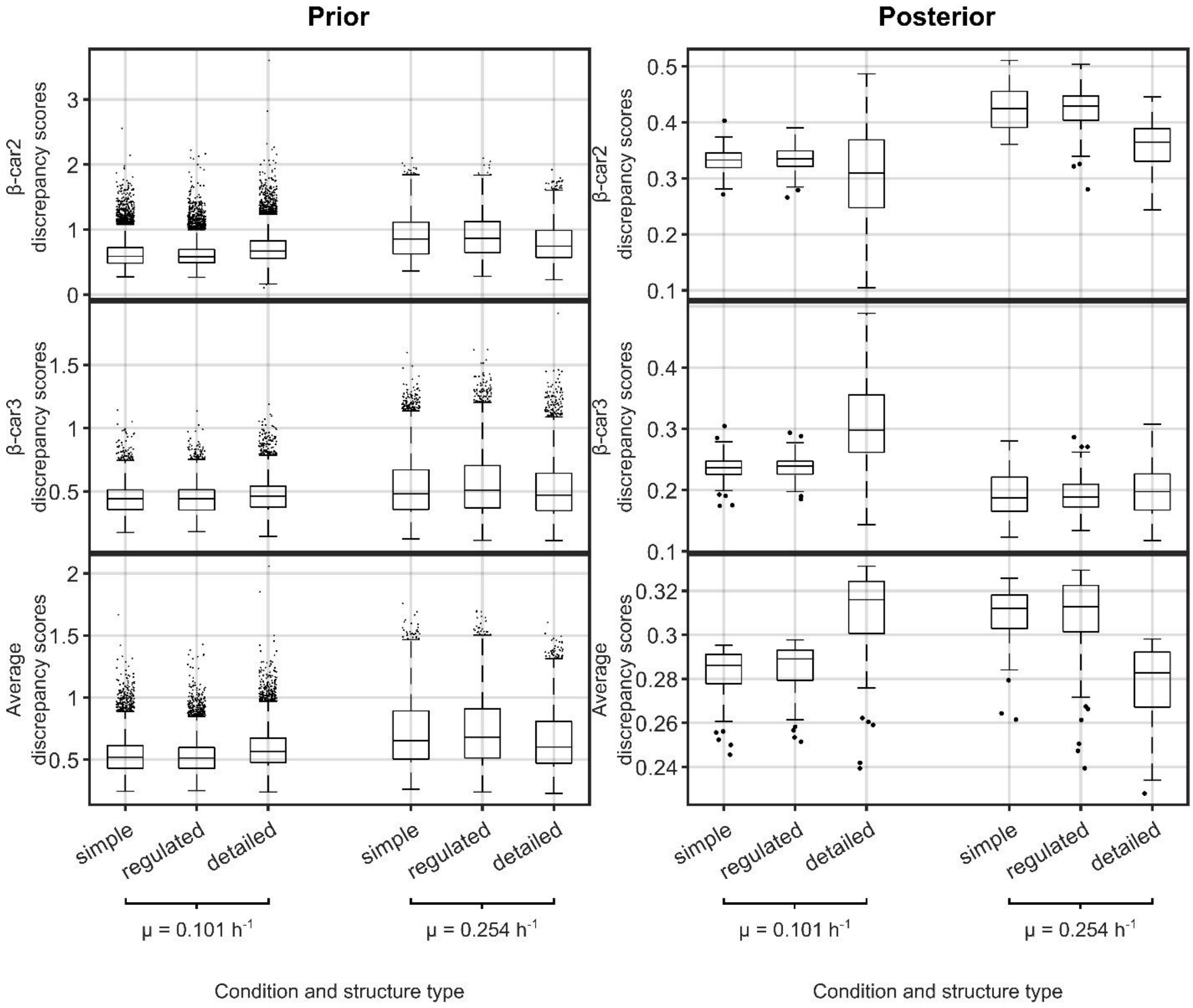

Figure S1. Distribution of ABC-rejection discrepancy scores. The boxplots are divided into the prior (left) and posterior (right) model particles. The discrepancy scores of the adjusted conditions β-car2, β-car3, and the average between them are displayed for each model particle. Within each boxplot, the discrepancy scores are separated by the ensemble of models generated for a specific growth rate and model structure.

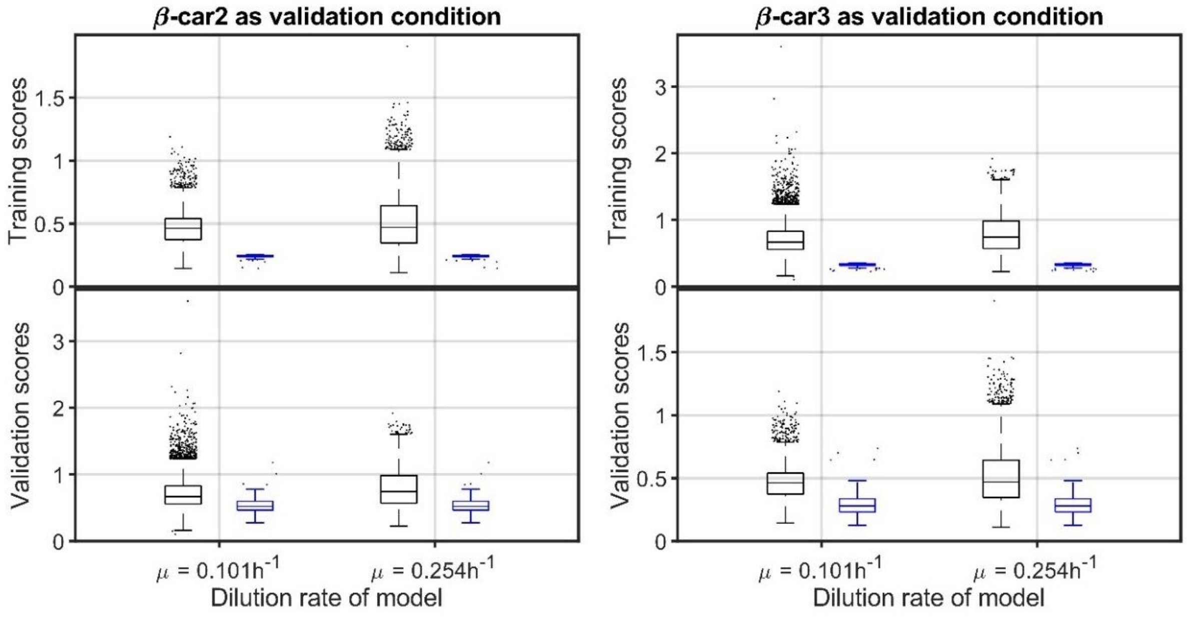

Figure S2. Cross-validation of discrepancy scores of the detailed models. The boxplots of discrepancy scores are divided according to the strain that was used for training (rejection) and for validation. The boxplots that used β-car3 for training and β-car2 for validation are located on the left, whereas the boxplots that used β-car2 for training and β-car3 for validation are located on the right. The boxplots located at the top are the discrepancy scores of the strain used for training, whereas the boxplots located at the bottom are the discrepancy scores of the strain used for validation. The ensembles sampled for low and high dilution rate are included in each case. The boxplots, lines, and dots in black represent prior discrepancy scores, whereas the boxplots, lines, and dots in blue correspond to the discrepancy scores of the trained ensembles. The validation scores were calculated as indicated in the development of posterior ensembles.

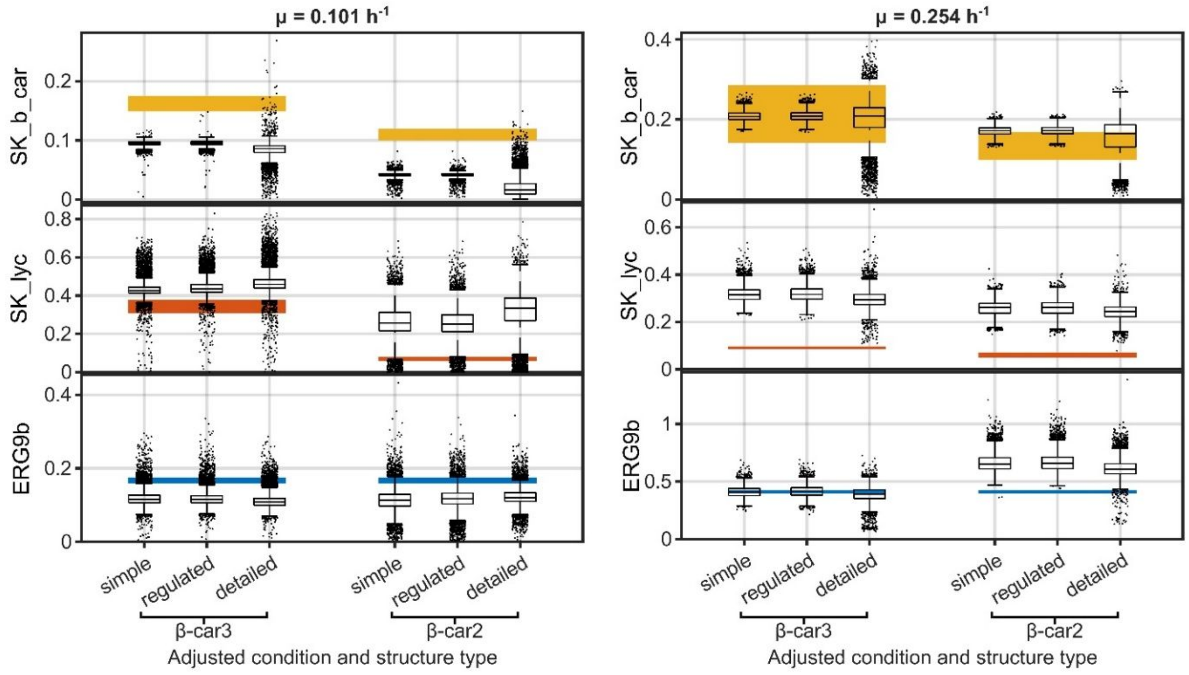

Figure S3. Prior flux distribution boxplots. The simulated flux distribution of reactions SK_b_car, SK_lyc, and ERG9b for the two growth rates studied are displayed. Each boxplot displays the simulated fluxes for the ensembles according to the model structure and the simulated strain (β-car2 and β-car3). The solid rectangles represent the 95% confidence interval of the corresponding experimentally measured flux. All fluxes are presented in mmol/L/h.

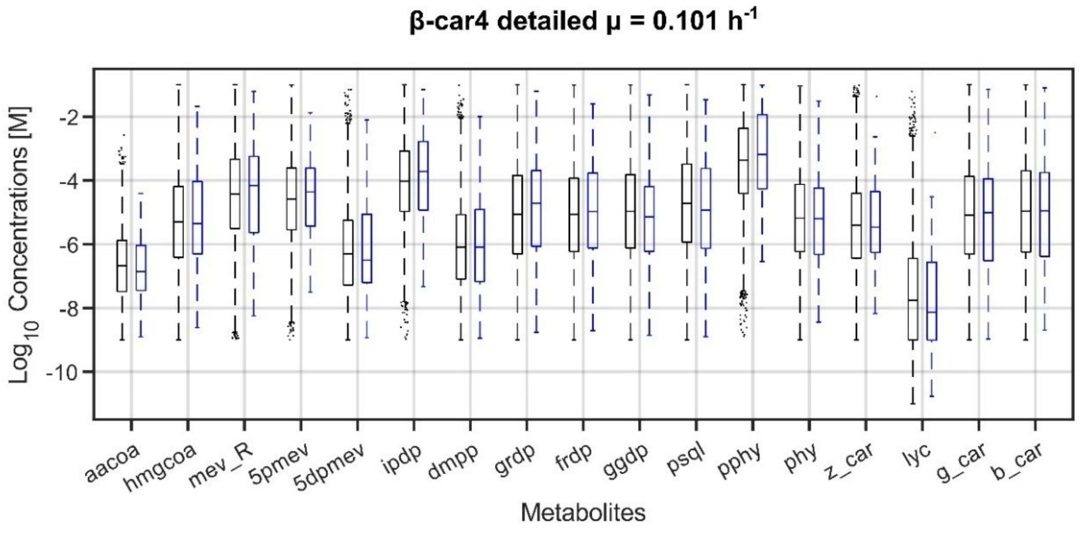

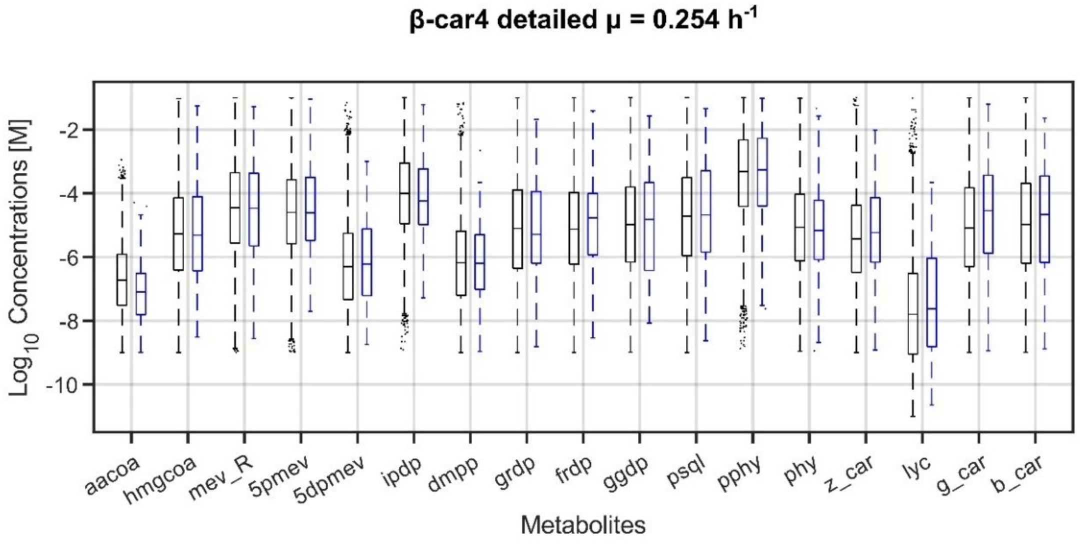

Figure S4. Prior and posterior metabolite concentration distribution boxplots. Each boxplot shows the metabolite concentrations of the model particles sampled using the β-car4 strain as reference. The boxplots depict the model ensembles with detailed structure generated at low (top) and high (bottom) growth rates. Black boxes, lines, and dots represent the prior model ensembles, whereas blue boxes, lines, and dots represent the posterior ensembles (after the rejection).

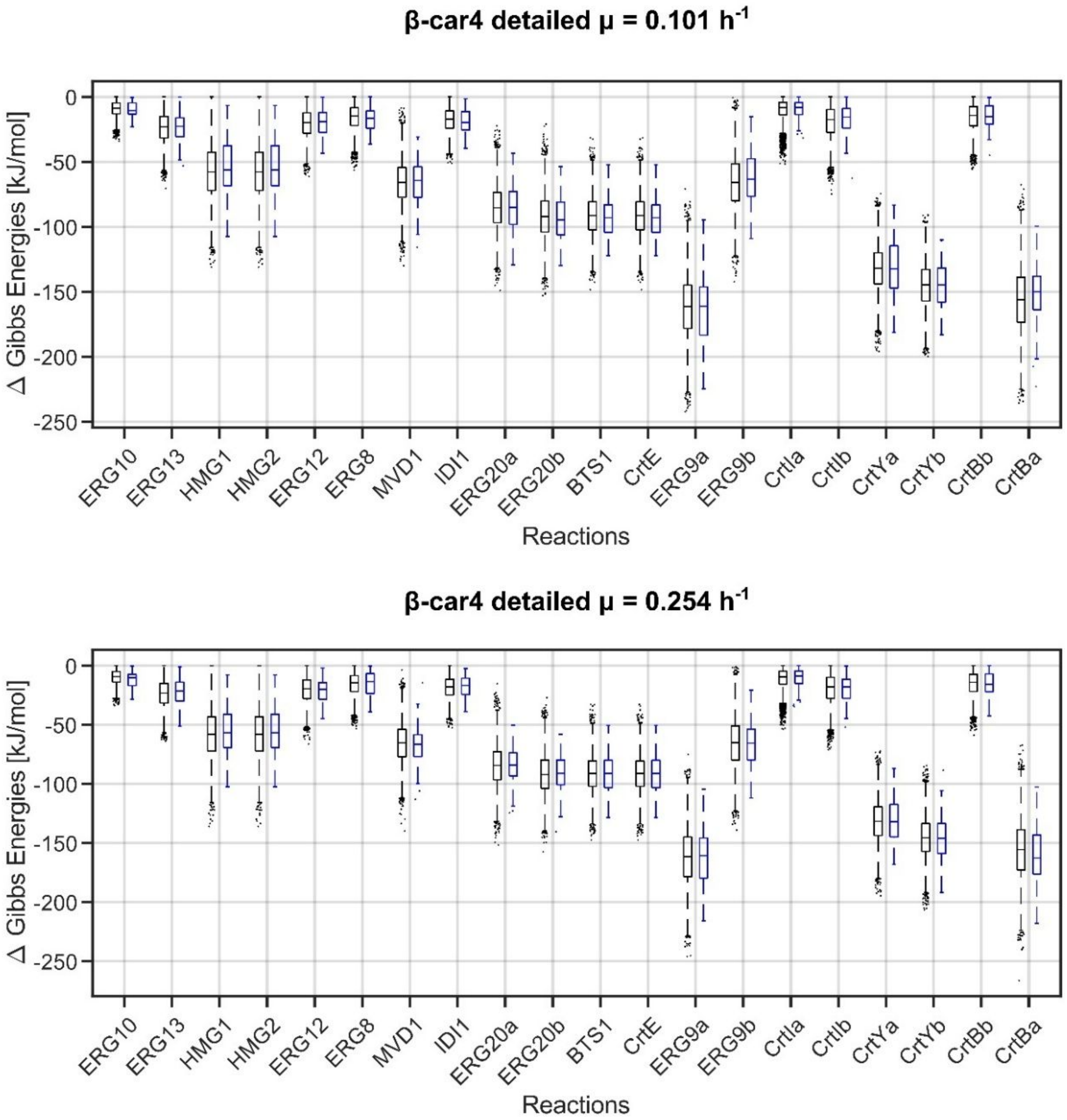

Figure S5. Prior and posterior Gibbs free energy of reactions distributions. Each boxplot shows the Gibbs free energies of model particles sampled for strain β-car4. The boxplots depict the model ensembles with detailed structure generated at low (top) and high (bottom) growth rates. Black boxes, lines, and dots represent the prior ensembles of models, whereas blue boxes, lines, and dots represent the posterior ensembles (after the rejection).

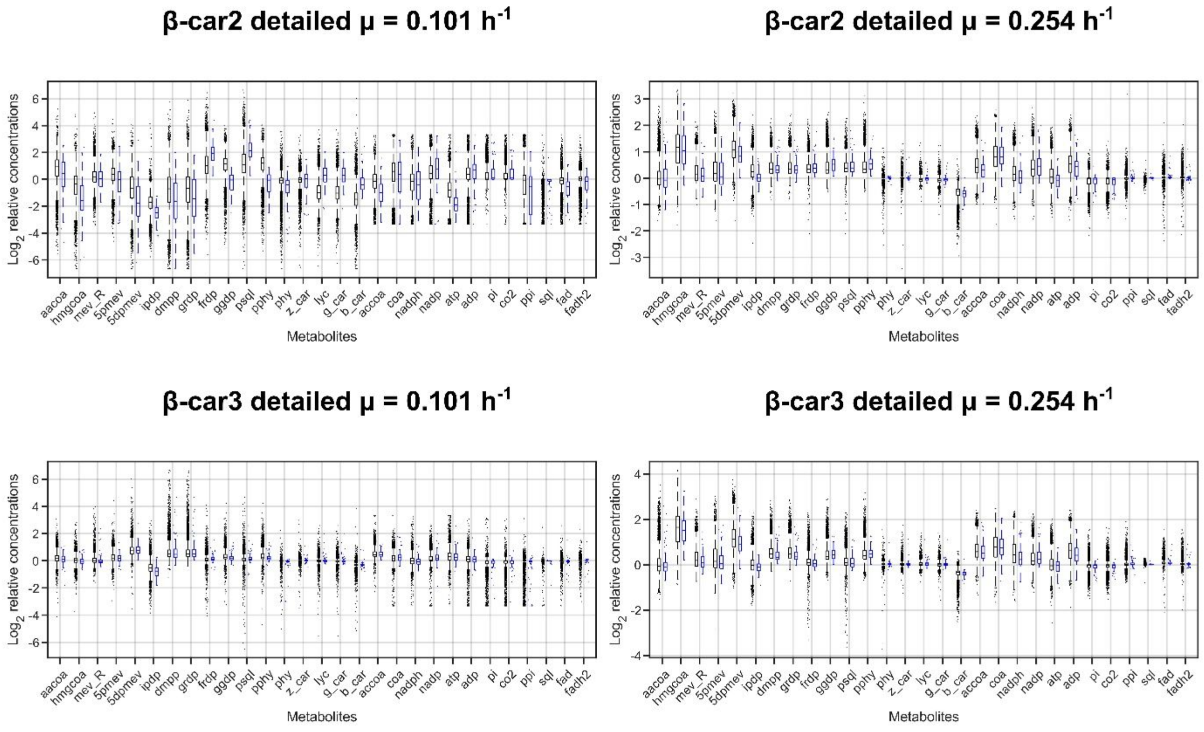

Figure S6. Boxplots of relative metabolite concentrations adjusted to the experimental training conditions using the detailed model ensemble. The figure is divided according to non-reference condition, β-car3 is located at the top, whereas β-car2 is located at the bottom. The dilution rates are divided in two, to the left is the low dilution rate and to the right is the high dilution rate. Each boxplot contains the distributions of the prior ensemble (black boxes, lines, and dots) and the posterior ensemble (blue boxes, lines, and dots) resulting from the rejection process. Relative quantities are presented on a logarithm scale based on the corresponding reference condition.

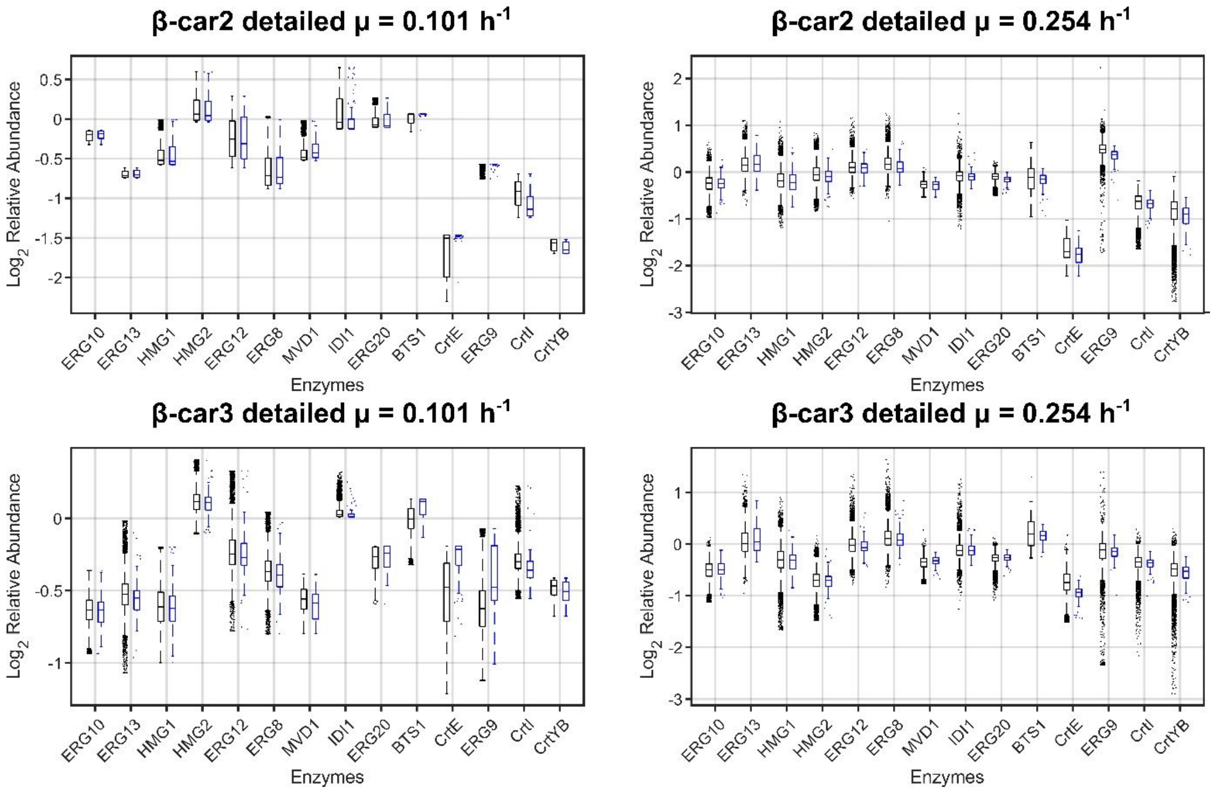

Figure S7. Boxplots of relative enzyme abundances adjusted to the experimental training conditions using the detailed model ensemble. The figure is divided according to non-reference condition, β-car3 is located at the top, whereas β-car2 is located to the bottom. The dilution rates are divided in two, to the left is the low dilution rate and to the right is the high dilution rate. Each boxplot contains the distributions of the prior ensemble (black boxes, lines, and dots) and the posterior ensemble (blue boxes, lines, and dots) resulting from the rejection process. Relative quantities are presented on a logarithm scale based on the corresponding reference condition.

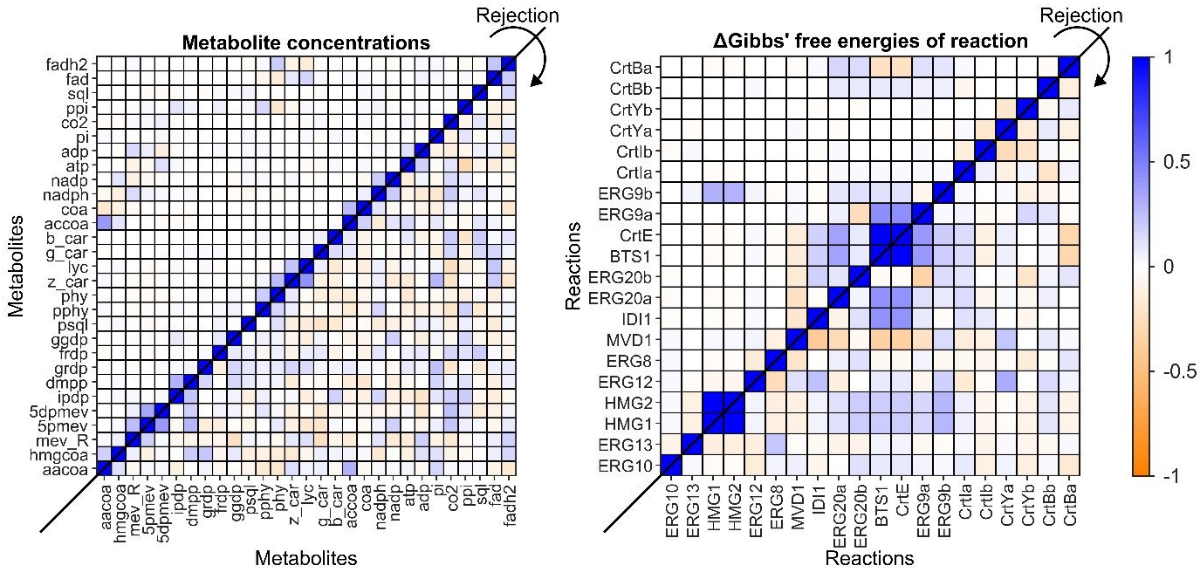

Figure S8. Heatmaps of Spearman rank correlation for metabolite concentrations and Gibbs free energies at dilution rate of 0.254 h^{-1}. The heatmaps summarize the change in spearman rank correlation of the metabolite concentrations (left) and $\Delta_r G$ (right) before and after the rejection of the detailed models. In each heatmap, the correlations of the prior ensembles are included in the upper triangular matrix, while the correlations of the posterior ensembles are presented in the lower triangular matrix. As for the case when dilution rate is 0.101 h^{-1}, there is a general enrichment in the correlations of the pathway metabolites and reactions.

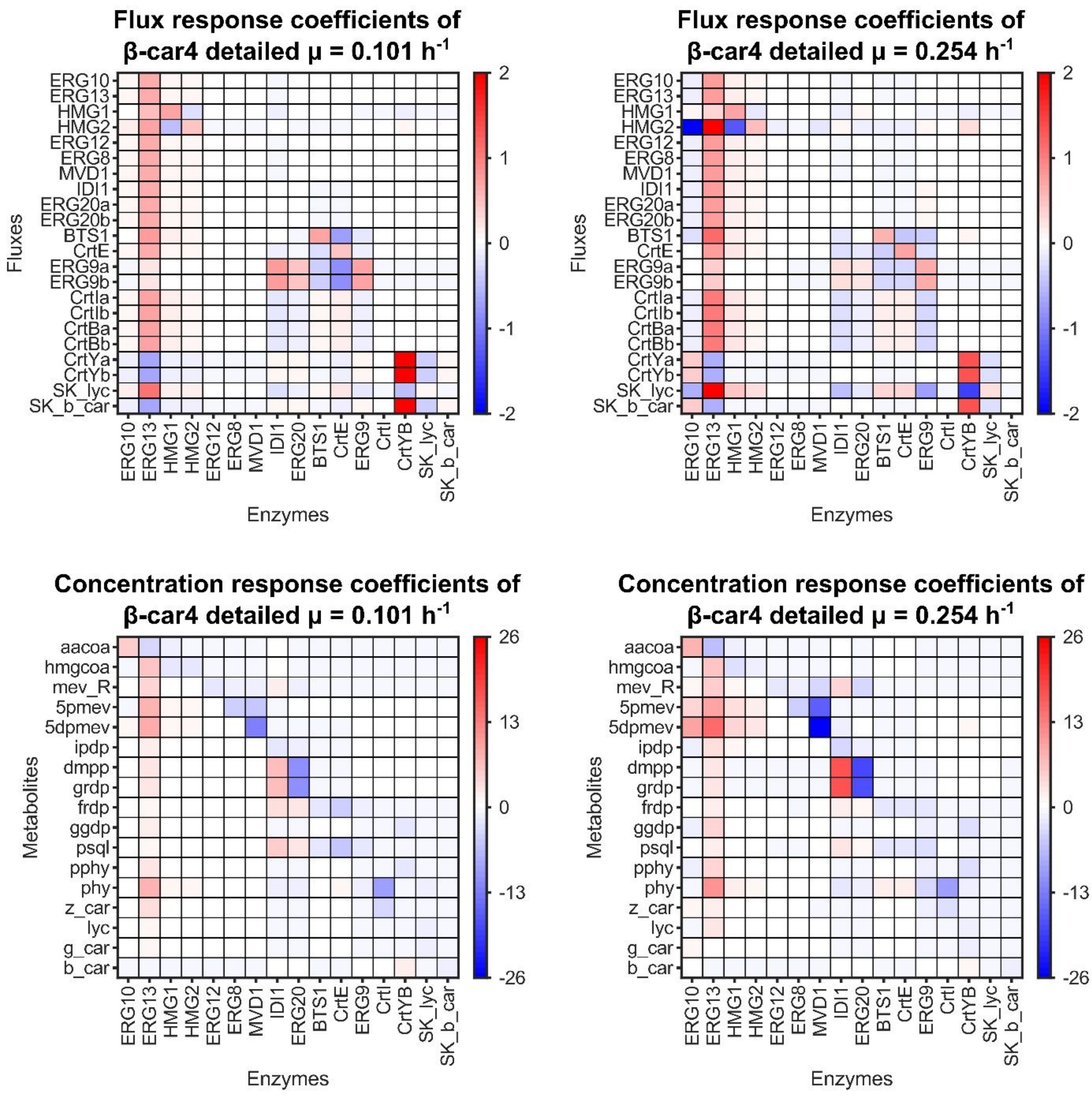

Figure S9. Mean response coefficients of the detailed models. Heatmaps portray the average response coefficients for the model ensemble with a detailed structure for the reference strain β-car4. Coefficients are divided into the growth rates used to generate and sample the model particles (left and right). Flux response coefficients are placed at the top, while concentration response coefficients are at the bottom.

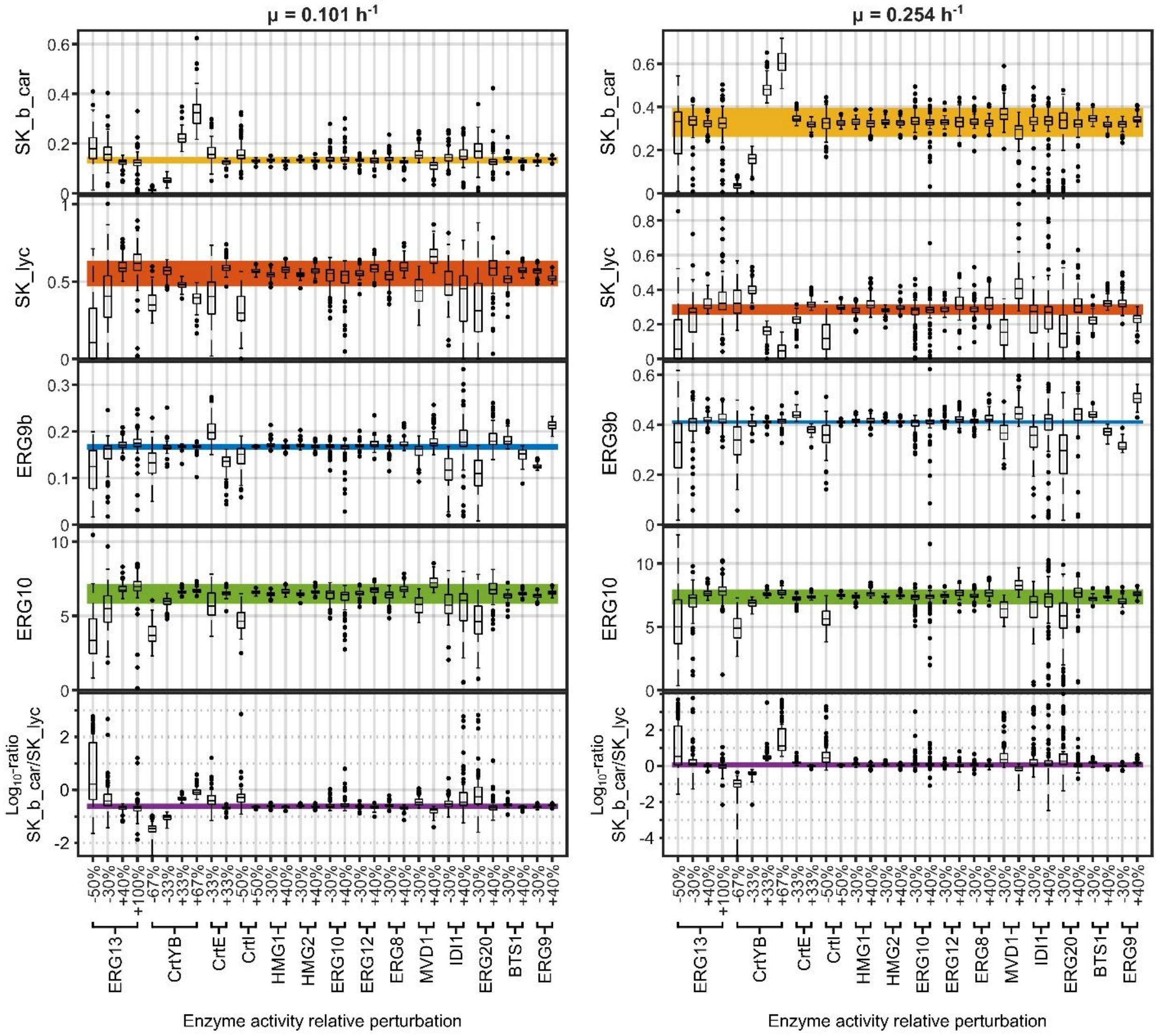

Figure S10. Simulated fluxes by the detailed ensembles under various enzyme perturbations. The simulated flux distribution of reactions SK_b_car, SK_lyc, ERG9b, and ERG10 for the two studied growth rates are displayed as well as the ratio between SK_b_car and SK_lyc. The figure shows the simulated fluxes for the model ensemble with a detailed structure at low (left) and high (right) dilution rates. Each boxplot depicts the fluxes under separate perturbations of enzyme activity levels relative to the reference strain β-car4. The solid rectangles represent the 95% confidence interval of the corresponding experimentally measured flux for the reference strain. All fluxes are presented in mmol/L/h. The SK_b_car/SK_lyc ratios are presented in Log10 scale, and its solid line is the mean ratio at the reference strain.

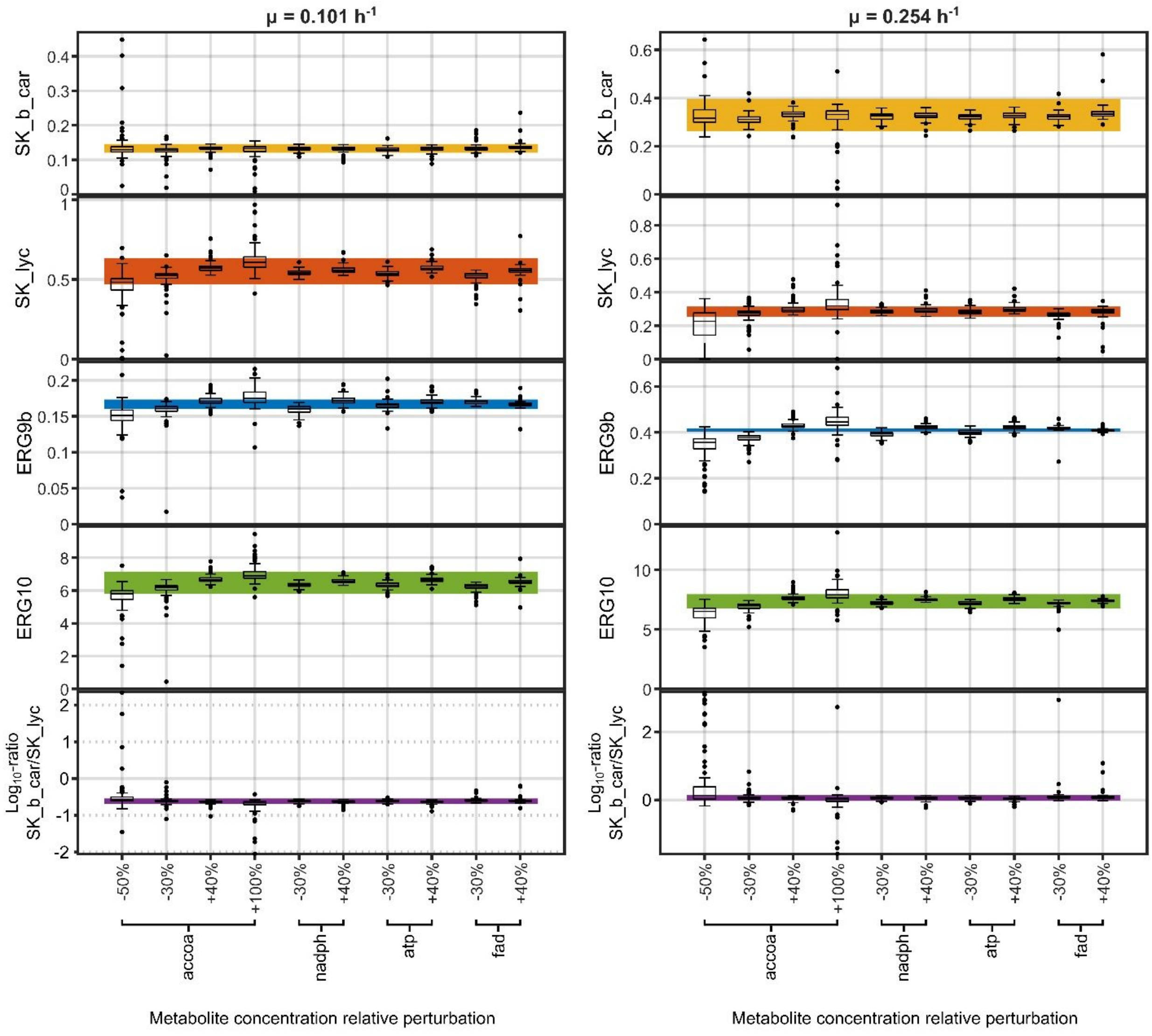

Figure S11. Simulated fluxes by the detailed ensembles under various metabolite perturbations. The simulated flux distribution of reactions SK_b_car, SK_lyc, ERG9b, and ERG10 for the two studied growth are displayed along the ratio between SK_b_car and SK_lyc. The figure shows the simulated fluxes for the model ensemble with a detailed structure at low (left) and high (right) dilution rates. Each boxplot depicts the fluxes under separate perturbations of non-balanced metabolite concentrations relative to the reference strain β-car4. The solid rectangles represent the 95% confidence interval of the corresponding experimentally measured flux at the reference strain. All fluxes are presented in mmol/L/h. The SK_b_car/SK_lyc ratios are presented in Log10 scale, and its solid line is the mean ratio at the reference strain.

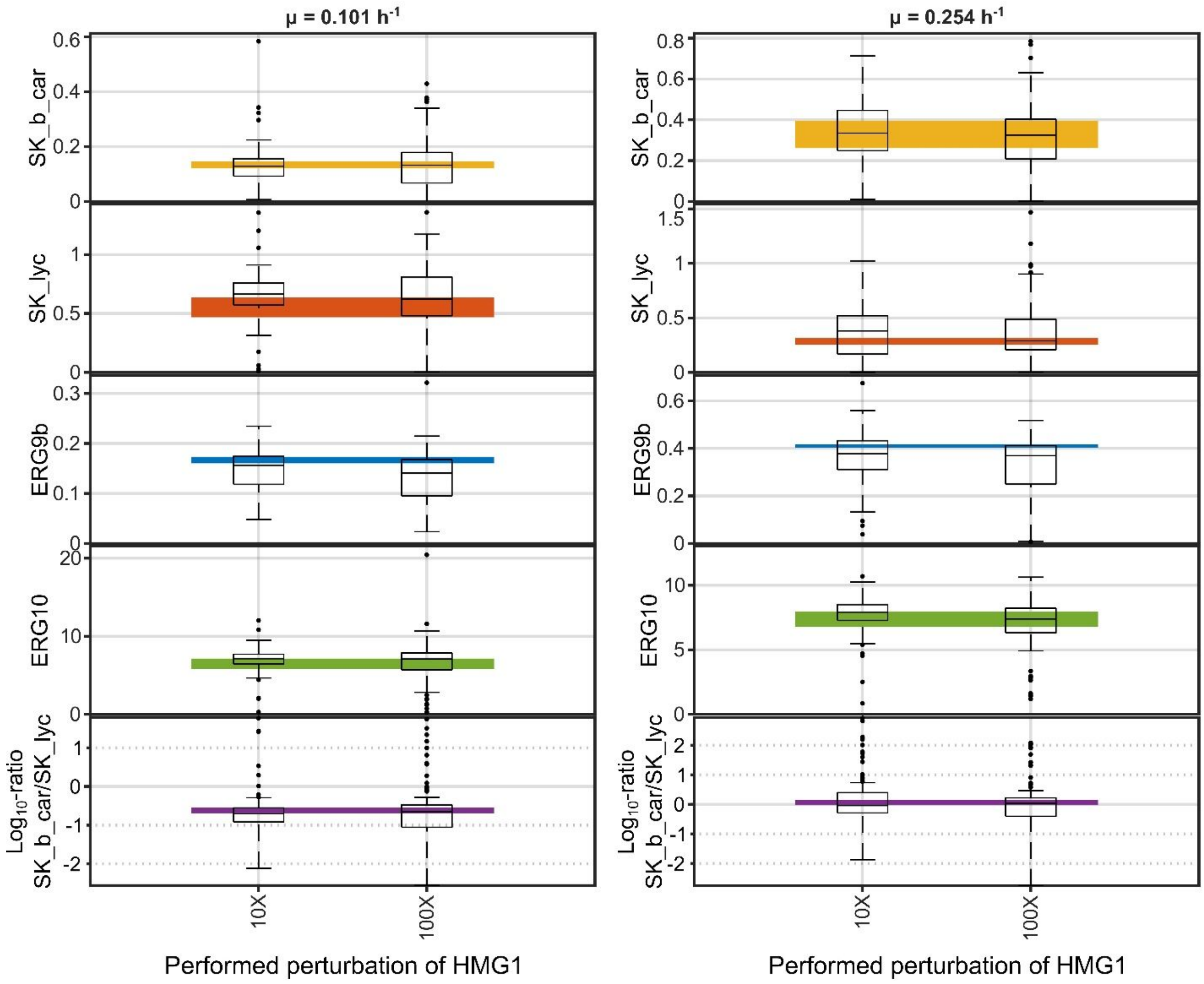

Figure S12. Simulated fluxes by the detailed ensembles upon the inclusion of the tHMG1 enzyme described by the upregulation of HMG1. The simulated flux distribution of reactions SK_b_car, SK_lyc, ERG9b, and ERG10 for the two dilution rates are displayed along the ratio between SK_b_car and SK_lyc. The figure shows the simulated fluxes for the model ensemble with a detailed structure at low (left) and high (right) growth rates. In both cases, 10-fold and 100-fold increases in the concentration of HMG1 compared to the reference concentration were tested. The solid rectangles represent the 95% confidence interval of the corresponding experimentally measured flux at the reference strain. All fluxes are presented in mmol/L/h. The SK_b_car/SK_lyc ratios are presented in Log10 scale, and its solid line is the mean ratio at the reference strain.

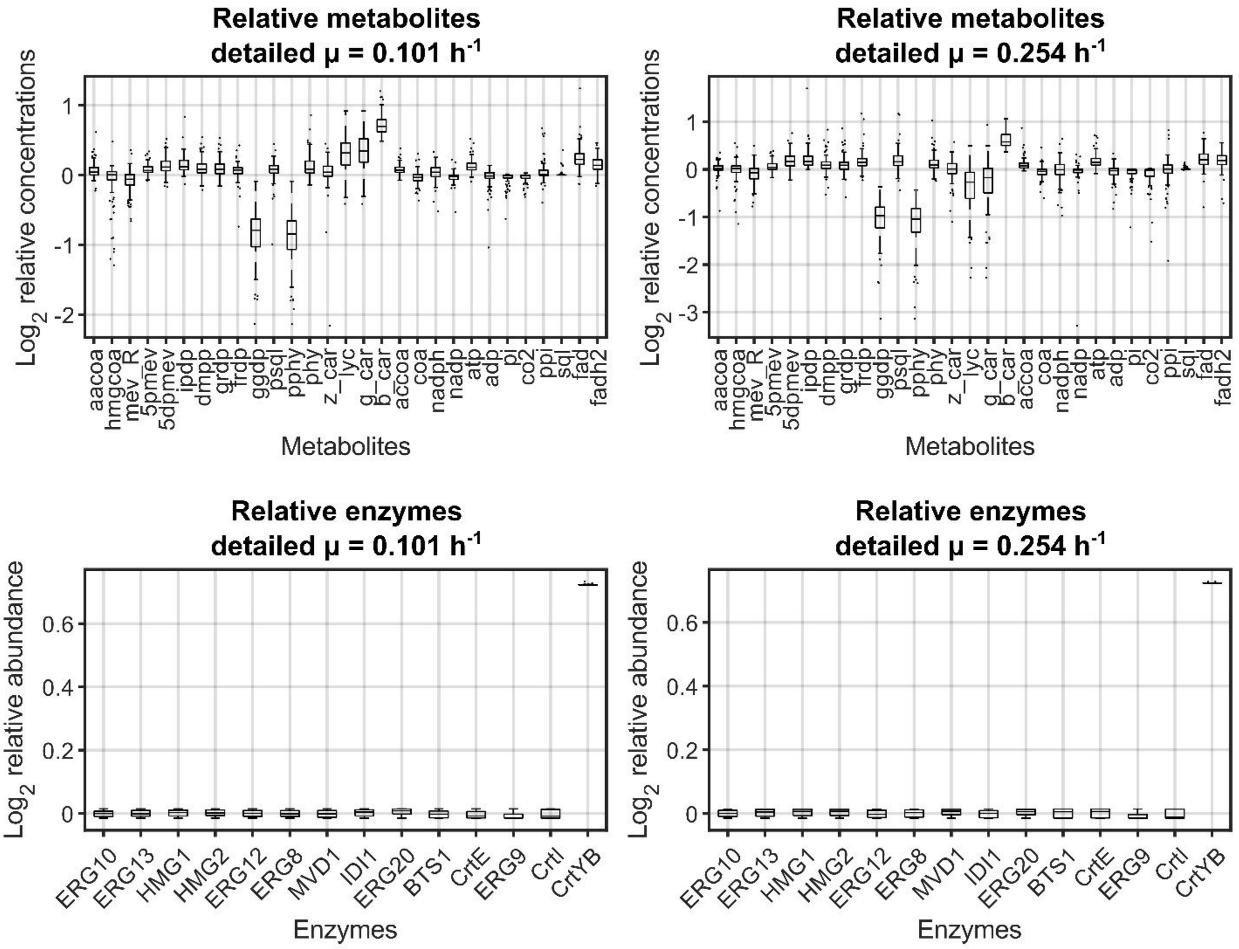

Figure S13. Relative metabolite and enzyme abundances under predicted operation conditions. The figure shows the boxplots of the estimated relative metabolite concentrations (top) and relative enzyme abundances (bottom) when a perturbation of a 67% increase in the CrtYB enzyme activity is performed to the reference strain β-car4. The boxplots depict the model ensembles with a detailed structure generated at low (left) and high (right) growth rates. All relative values are in Log2 scale.

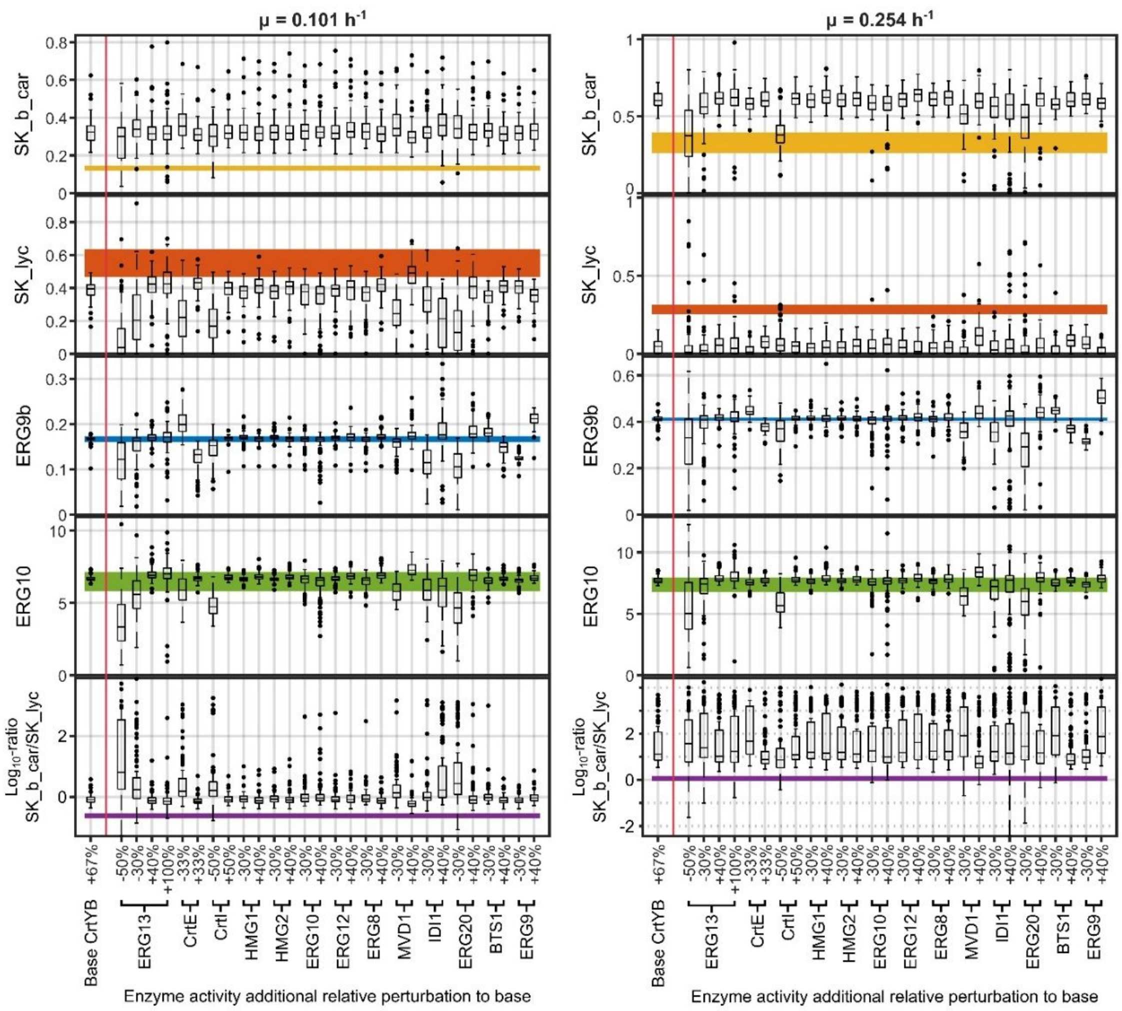

Figure S14. Simulated fluxes by the detailed ensembles with additive enzyme perturbations. The simulated flux distribution of reactions SK_b_car, SK_lyc, ERG9b, and ERG10 under the two dilution rates are displayed along the ratio between SK_b_car and SK_lyc. The figure shows the simulated fluxes for the model ensembles with a detailed structure at low (left) and high (right) growth rates. Each boxplot depicts the fluxes under a perturbation increasing 67% the enzyme activity of CrtYB, with additive perturbations of enzyme activity levels relative to the reference strain β-car4. Base CrtYB is the case that includes the CrtYB change without additional perturbations. The solid rectangles represent the 95% confidence interval of the corresponding experimentally measured flux for the reference strain. All fluxes are presented in mmol/L/h. The SK_b_car/SK_lyc ratios are presented in Log10 scale, and its solid line is the mean ratio at the reference strain.

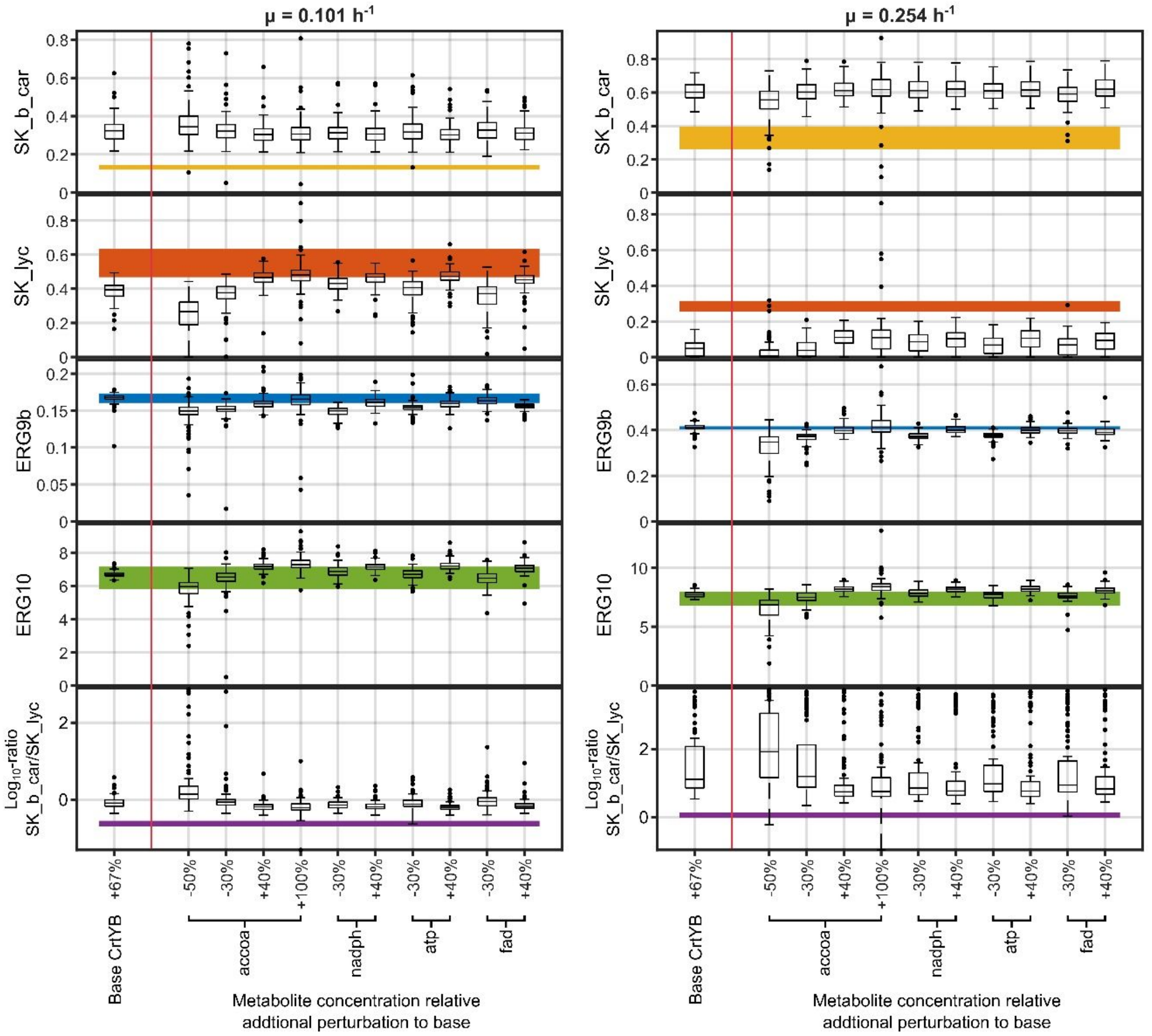

Figure S15. Simulated fluxes by the detailed ensembles with additive metabolite perturbations. The simulated flux distribution of reactions SK_b_car, SK_lyc, ERG9b, and ERG10 for the two studied growth are displayed along the ratio between SK_b_car and SK_lyc. The figure shows the simulated fluxes for the model ensembles with a detailed structure at low (left) and high (right) growth rates. Each boxplot depicts the fluxes under a perturbation increasing 67% the enzyme activity of CrtYB, with additive perturbations of non-balanced metabolite concentrations relative to the reference strain β-car4. Base CrtYB is the case that includes the CrtYB change without additional perturbations. The solid rectangles represent the 95% confidence interval of the corresponding experimentally measured flux for the reference strain. All fluxes are presented in mmol/L/h. The SK_b_car/SK_lyc ratios are presented in Log10 scale, and its solid line is the mean ratio at the reference strain.

III. Supplementary Methods

S1. Estimation of dry cell weight

A conversion factor between OD600nm and dry cell weight (DCW) concentration was estimated for each of the strains in the study. Each strain was cultivated overnight in shake flasks with a working volume of 250mL using the same media as chemostat cultivations. Flask cultivations of each strain were executed in 4 independent replicates. During the late exponential growth phase, part of the culture was used for performing OD600nm measurements and drying cell mass. OD600nm measurements were carried out in triplicates. Samples were diluted to fall within the linear range of the OD600nm versus biomass concentration relationship. The remaining volume of the flask fermentation was used to estimate dry cell weight concentration. The biomass was recovered using a Büchner funnel with quantitative filter paper. Then, the paper with the biomass was dried, thereby obtaining the total dry cell weight. The conversion factor of each shake flask replicate was estimated by dividing the dry cell weight concentration by the corresponding OD600nm average.

Conversion factors of each strain were checked for outliers using the median criterion included in MATLAB R2021a[13]. Outliers from the replicates were eliminated, and the rest were used to estimate the mean conversion factor of the strain. It was assumed that the mean factors followed a t-distribution. Table S7 summarizes the obtained conversion factors.

Table S7. Conversion factors of dry cell weight concentration and OD600nm measurements.

Strain	Mean [(gDCW/L)/ OD600]	SD [(gDCW/L)/ OD600]	SE [(gDCW/L)/ OD600]	Measurements	Degrees of Freedom (DoF)
β-car2	0.533	0.017	0.0085	4	3
β-car3	0.541	0.004	0.0021	3	2
β-car4	0.498	0.012	0.0062	4	3

S2. Chemostats operation

The dilution rates of the chemostat cultivations were estimated based on the working volume and the medium feeding rate controlled by a peristaltic pump (120U/D1 model, Watson-Marlow, UK). The bioreactors included an outlet duct located at the height where the desired working volume was met. An outlet pump connected to the exit duct operated at a higher flow rate than the feeding pump to maintain a constant working volume. Since pumps used

revolutions per minute (RPM) as control input variable, a linear regression model was used to estimate the feeding flow rate as a function of RPM. The mean feeding flow rate was assumed to follow a *t*-distribution. The results for the two dilution rates are summarized in Table S8. Here, the dilution rates are equivalent to the specific growth rates since the chemostats operate at steady state.

Table S8. Dilution rates of the chemostat cultivations

Dilution Rate	Mean (1/h)	SE (1/h)	Measurements	DoF	Lower 95% CI (1/h)	Upper 95% CI (1/h)
Low	0.101	0.00157	8	6	0.0971	0.1048
High	0.254	0.00149	8	6	0.2507	0.2580

Several OD600nm measurements were performed during chemostats cultivations at steady state. Outliers were identified and eliminated following the median criterion included in MATLAB R2021a[13]. The average OD600nm of the chemostats were assumed to follow a *t*-distribution. The results are shown in Table S9.

Table S9. Measured OD600nm of the chemostats at steady state.

Strain	Growth rate (1/h)	Mean OD600	SD OD600	SE OD600	Measurements	DoF
β-car2	0.101	0.812	0.013	0.003	15	14
β-car2	0.254	0.346	0.003	0.001	12	11
β-car3	0.101	0.695	0.007	0.002	15	14
β-car3	0.254	0.179	0.005	0.001	15	14
β-car4	0.101	1.104	0.023	0.005	19	18
β-car4	0.254	0.307	0.008	0.002	18	17

The estimated parametric distributions of the conversion factors, dilution rates and OD600nm of the chemostats were used to calculate the metabolic fluxes of the strains under the studied growth conditions.

S3. Reverse transcription quantitative Polymerase chain reaction (RT-qPCR)

The procedure from the generation of RNA samples in chemostat cultivations to the qRT-PCR quantification was performed within a year. Samples treated with RNA Save Solution (Biological Industries, Israel) were maintained at -80°C before performing the consecutive steps for their analysis.

- RNA extraction

The entire 15mL samples were used for RNA extraction by separate. After thawing the samples in ice, the E.Z.N.A® Total RNA Kit I (OMEGA Bio-Tek, GA, USA) was used for extraction employing the Centrifugation and DNase I Digestion Protocol recommended by the producer. The complete protocol was performed in a laminar flow cabinet previously cleansed with RNAseZap® Solution (AB Applied Biosystems, MA, USA) to prevent RNAse contamination. 2-mercaptoethanol purchased from Sigma-Aldrich (MA, USA) was included in the lysis buffer as suggested by the protocol. An additional homogenization step was required at the beginning of extraction. The step consisted of disrupting the cells resuspended in the TRK Lysis Buffer from the kit in a homogenizer. Cells were disrupted with 0.5mL of 0.5-mm RNAse-free glass beads purchased from OMEGA Bio-Tek for 10 minutes at 3500 rpm. Samples were eluted from the RNA binding column using 50µL of RNAse-free water. Then RNA concentration and purity were quantified using Nanodrop™ (Thermo Fisher Scientific) in triplicate, average measurements are included in Table S10. All RNA samples were stored immediately at -80°C for later processing.

Table S10. Extracted RNA concentration and purity measurements.

Strain	Growth rate (1/h)	Extraction replicate	Average RNA concentration (ng/µL)	Average A260/A230	Average A260/A280
β-car2	0.101	1	531.40	2.37	2.08
β-car2	0.101	2	549.43	2.41	2.08
β-car2	0.101	3	506.50	2.40	2.08
β-car2	0.254	1	279.80	2.09	2.13
β-car2	0.254	2	100.57	1.74	2.12
β-car2	0.254	3	96.43	1.98	2.08
β-car3	0.101	1	534.03	2.34	2.08
β-car3	0.101	2	427.17	2.44	2.11
β-car3	0.101	3	523.10	2.41	2.08
β-car3	0.254	1	93.73	1.92	2.07
β-car3	0.254	2	90.90	2.11	2.13
β-car3	0.254	3	53.40	2.20	2.10
β-car4	0.101	1	465.80	2.35	2.10
β-car4	0.101	2	523.20	2.36	2.09
β-car4	0.101	3	461.10	2.47	2.10
β-car4	0.254	1	124.57	2.11	2.10
β-car4	0.254	2	234.73	2.41	2.15
β-car4	0.254	3	103.43	2.17	2.11

Integrity and DNA contamination were checked by denaturing RNA electrophoresis as explained by Masek[14] with 0% formamide. Ethidium bromide was replaced with SYBR Safe gel stain (Thermo Fisher Scientific, MA, USA). 10µL of each sample were mixed with 2µL of 6X Purple Gel Loading Dye (New England Biolabs, MA, USA) before loading to the agarose gel. Negative controls with nuclease-free water were used to check for possible contamination of the assays. RNA bands were visualized in a GelDoc Go System (Bio-Rad, CA, USA). In all cases rRNA 18S and 25S bands were visible, whereas DNA contamination and RNA degradation were not observed.

- Reverse transcription

cDNA was generated from the extracted RNA samples using the AffinityScript QPCR cDNA Synthesis kit (Agilent Technologies, CA, USA) with Oligo-dT primers. The composition of each separate reaction is included in Table S11. For each reaction a No-RT (no reverse transcription) control was performed to then be included in qPCR. The reactions were set-up on ice inside a laminar flow cabinet previously cleansed with RNAseZap® Solution (AB Applied Biosystems) and DNA-OFF™ (Takara Bio USA, CA, USA). The thermocycler program used for reverse transcription is portrayed in Table S12. cDNA samples were then diluted to 1/10 of the original volume for qPCR procedures and stored at -20°C.

Table S11. Reverse transcription reaction composition.

Reaction component	RT reaction	No-RT reaction
RNAse-free water	Up to 45µL	Up to 15µL
First strand mastermix	22.5µL	7.5µL
Oligo(dT) primers	6.75µL	2.25µL
AffinityScript RT/RNase Block enzyme mixture	2.25µL	0.75µL
Sample RNA	2.25µg (or 13.5µL from the sample if 2.25µg could not be obtained from that volume)	0.75µg (or 4.5µL from sample the if 2.25µg could not be obtained from that volume)

Table S12. Thermocycler program for reverse transcription.

Segment	Number of cycles	Temperature	Duration
1	1	25°C	5 minutes
2	1	42°C	5 minutes
3	1	55°C	15 minutes
4	1	95°C	5 minutes
5	1	4°C	(To remove samples)

- Primers design

Each cDNA sample was used for qPCR measurements of all the genes in the kinetic model of β-carotene metabolism. Four additional genes (ALG9, TAF10, UBC6, and ACT1) were included to later be used as references when relativizing transcript abundances. The use of genes ALG9, TAF10, and UBC6 was based on the study of Teste[15]. ACT1 was included because it is a widely used housekeeper gene[16–19]. Specificity of the target sequences was tested *in silico* while designing primers in the Primer-BLAST tool provided by the National Center for Biotechnology Information[20]. Secondary structure evaluation of amplicons was done *in silico* in the latter platform and in the OligoAnalyzer™ Tool[21] from Integrated DNA Technology (NJ, USA). Primers were manufactured and purified using a desalting technique by Macrogen (South Korea). Table S13 includes the gene symbols of target amplicons, Genbank[22] mRNA accession number, amplicon length, primer sequences, and sequence alignment.

Table S13. Summary of gene target amplicons and primers used for RT-qPCR.

Gene	GenBank mRNA Accession number	mRNA length	Amplicon Length	Primers 5'-3' sequence	Strand	Alignment start	Alignment end
ERG10	NM_001183842.1	1197	113	GGGCTCCATCTCTTGCAGTT	Plus	884	903
				GTTCACCAAACCGACAACCG	Minus	996	977
ERG13	NM_001182489.1	1476	116	GGTGCCGGTACTGTTGCTAT	Plus	607	626
				TCGCTGGTGAAATCTGGCTT	Minus	722	703
HMG1	NM_001182434.1	3165	125	TGGTAAGAGTGTCGTCGCAG	Plus	2577	2596
				CAGCCATTGCAGATCCAACC	Minus	2701	2682
HMG2	NM_001182338.1	3138	102	ATGGCTGGATCTGTTGGTGG	Plus	2683	2702
				GGAACTTTCGACGTTCTGCG	Minus	2784	2765
ERG12	NM_001182715.1	1332	141	TGCCATCACCGAGGATCAAG	Plus	207	226
				AACGCTGCATGGTAGTGGAA	Minus	347	328
ERG8	NM_001182727.1	1356	180	CCTCGGCAGGTTTAGTCACA	Plus	470	489
				TAGATCCATATGCTGCCGCC	Minus	649	630
MVD1	NM_001183220.1	1191	153	GCTCTGTTCCTGGATGGGAC	Plus	983	1002
				GGCCTGAACCGACTTGAGTT	Minus	1135	1116
IDI1	NM_001183931.1	867	125	ATTACTGCGGCGGTGAGAAA	Plus	487	506
				CCCCATGGTTCATTGCTTGG	Minus	611	592
ERG20	NM_001181600.1	1059	121	ACTCAGTCGCAGAAGCCAAAT	Plus	872	892
				TCGTCGACTTGGGAGATCTTG	Minus	992	972
ERG9	NM_001179321.1	1335	158	TCCAGATCGTTTGCTGCTGT	Plus	148	167
				TTCTCGTGGAAGTGACGCAA	Minus	305	286
BTS1	NM_001183883.1	1008	141	ATGGAAGCGCTGTCTCCTTC	Plus	541	560
				CTCAGCAAAGCCTTTTTCGCT	Minus	681	661
CRTE	MZ748349.1	1131	195	CTCGATTCATGCCAACCCCT	Plus	828	847
				GCGAACTCTCCTTGAAGCCT	Minus	1022	1003
CRTI	MZ748351.1	1749	98	GAAGCATCTTGGGACTGGCT	Plus	1385	1404
				AGCCCCGACAAAGAACAACT	Minus	1482	1463

Table S13 (continued). Summary of gene target amplicons and primers used for RT-qPCR.

Gene	GenBank mRNA Accession number	mRNA length	Amplicon Length	Primers 5'-3' sequence	Strand	Alignment start	Alignment end
CRTYB	MZ748350.1	2022	177	TGTTAGTGGCAAGCCGAGAG	Plus	1526	1545
				CGAAATCTTGAGGCCGAGGT	Minus	1702	1683
TAF10	NM_001180474.3	621	195	GGATCAGGTCTTCCGTAGCG	Plus	398	417
				TGAGCCCGTATTCAGCAACA	Minus	592	573
UBC6	NM_001178991.3	753	117	GGATGCGGCAAATACAGGTG	Plus	519	538
				GCTTGTTCAGCGCGTATTCT	Minus	635	616
ALG9	NM_001183057.1	1668	96	CCATCAGAACCGCATTCGAC	Plus	620	639
				CAGGAGCAAGCTTCCCGTAA	Minus	715	696
ACT1	NM_001179927.1	1083	139	AGTTGCCCCAGAAGAACACC	Plus	240	259
				GGACAAAACGGCTTGGATGG	Minus	378	359

- qPCR assays

qPCR assays were conducted in an AriaMx Real-time PCR System (Agilent Technologies, CA, USA) using the Brilliant II SYBR Green QPCR Master Mix (Agilent Technologies). 96-well plates, non-skirted low profile (Agilent Technologies) sealed with AriaMx adhesive plate seals (Agilent Technologies) were used for the assays. All plates were prepared manually inside a laminar flow cabinet cleansed with DNA-OFF™ (Takara Bio USA). Each reaction had a volume of 12.5µL and the composition of them are included in Table S14. The thermocycler program used for the assays is depicted in Table S15.

Table S14. qPCR reaction composition.

Reaction component	Sample reaction	No-RT reaction	Standard reaction	No template control reaction
2x Brilliant II SYBR Green QPCR Master Mix	6.25µL	6.25µL	6.25µL	6.25µL
Forward and Reverse primer mix (5µM each)	1µL	1µL	1µL	1µL
Template	2.5µL	2.5µL	2.5µL	0µL
Nuclease-free water	2.75µL	2.75µL	2.75µL	5.25µL

Table S15. Thermocycler program for qPCR reactions.

Segment	Number of cycles	Temperature	Duration
1	1	95°C	10 minutes
2	40	95°C	30 seconds
		60°C (Detect Fluorescence)	1 minute
3	1	95°C	30 seconds
		55°C (Detect fluorescence)	30 seconds
		95°C	30 seconds

One gene was measured per plate/assay. Three separate technical replicate reactions were performed for each of the three extraction replicates. Also, each extraction replicate had a No-RT control on the plate to account for DNA contamination. For the standard curve, each gene standard was serially diluted to generate dilutions ranging from 10^{-5} to 10^{-11} of the original titers. Three separate technical replicate reactions were used for each of the dilutions. Finally, three NTC (no

template control) reactions were used to examine contamination of the master mix. The planned distribution of samples, standards, and controls in the qPCR plates is included in Table S16.

Table S16. Distribution of samples, standards, and controls in qPCR plates. Samples are included in yellow and follow the nomenclature X.Y.Z. X stands for the strain of the sample (2=β-car2, 3=car3,4=β-car4), Y for the growth rate (A=low, B = high), and Z for the number of the extraction replicate. No-RT controls follow the same nomenclature as samples and are shown in blue. Standards are depicted in green and follow the nomenclature ST.X, where X is the number of the 1/10 serial dilution relative to the standard with highest concentration. NTCs are portrayed in grey.

Row/column	1	2	3	4	5	6	7	8	9	10	11	12
A	2.A.1	2.A.1	2.A.1	2.A.2	2.A.2	2.A.2	2.A.3	2.A.3	2.A.3	ST.1	ST.1	ST.1
B	2.B.1	2.B.1	2.B.1	2.B.2	2.B.2	2.B.2	2.B.3	2.B.3	2.B.3	ST.2	ST.2	ST.2
C	3.A.1	3.A.1	3.A.1	3.A.2	3.A.2	3.A.2	3.A.3	3.A.3	3.A.3	ST.3	ST.3	ST.3
D	3.B.1	3.B.1	3.B.1	3.B.2	3.B.2	3.B.2	3.B.3	3.B.3	3.B.3	ST.4	ST.4	ST.4
E	4.A.1	4.A.1	4.A.1	4.A.2	4.A.2	4.A.2	4.A.3	4.A.3	4.A.3	ST.5	ST.5	ST.5
F	4.B.1	4.B.1	4.B.1	4.B.2	4.B.2	4.B.2	4.B.3	4.B.3	4.B.3	ST.6	ST.6	ST.6
G	2.A.1	3.A.1	4.A.1	2.A.2	3.A.2	4.A.2	2.A.3	3.A.3	4.A.3	ST.7	ST.7	ST.7
H	2.B.1	3.B.1	4.B.1	2.B.2	3.B.2	4.B.2	2.B.3	3.B.3	4.B.3	NTC	NTC	NTC

Analysis of raw fluorometric data was performed in the Agilent AriaMx Software© (version 2.0, downloaded on March 1st, 2023) to obtain quantification cycles (C_q) of each of the qPCR samples. The C_q values were obtained in each assay for a threshold of relative fluorescence at the exponential phase. All products were checked for specificity using melting curves. The reactions that had a different melting curve for the amplicons were discarded. The MATLAB R2021a environment[13] was used to estimate the calibration curves of the standards. The linear relationships between standard dilution relative concentrations in log10 scale and C_q values were determined. The dilutions that did not belong to the linear range were discarded from the calibration curves. All the reactions from the extraction samples subjected to retro-transcription were within the linear range, and therefore, above the limit of detection according to the analysis. Table S17 contains the linear regression results and efficiency of the standards for each of the conducted assays. The lowest C_q values of the NTCs in each assay are also included. Figures S16, S17, and S18 portray the linear regressions with their 95% confidence bounds and the mean C_q values of the technical replicates along their 95% confidence intervals.

Table S17. Statistics of the standard curves for the qPCR assays. ND stands for not detected. Confidence intervals of the efficiencies were estimated by calculating the confidence intervals of the slopes and converting them to efficiency percentages.

Gene	Slope	Intercept	Adjusted R^2	Efficiency (%)	Efficiency Lower 95% CI	Efficiency Upper 95% CI	Lowest standard serial dilution in calibration curve	Highest standard serial dilution in calibration curve	Lowest NTC Cq
ERG10	-0.287	-0.621	0.9982	93.56	90.97	96.18	10^{-11}	10^{-5}	36.34
ERG13	-0.283	-0.661	0.9984	91.86	89.49	94.25	10^{-11}	10^{-5}	ND
HMG1	-0.291	-0.537	0.9986	95.23	92.74	97.76	10^{-10}	10^{-5}	ND
HMG2	-0.289	-0.648	0.9992	94.45	92.79	96.11	10^{-11}	10^{-5}	ND
ERG12	-0.287	-0.638	0.9985	93.58	91.17	96.03	10^{-11}	10^{-5}	ND
ERG8	-0.278	-1.226	0.9980	89.82	87.31	92.36	10^{-11}	10^{-5}	ND
MVD1	-0.279	-0.790	0.9980	90.09	87.50	92.72	10^{-11}	10^{-5}	ND
IDI1	-0.290	-0.658	0.9980	95.17	92.29	98.09	10^{-11}	10^{-5}	37.73
ERG20	-0.286	-0.762	0.9988	93.39	91.22	95.59	10^{-11}	10^{-5}	ND
ERG9	-0.285	-0.800	0.9976	92.53	89.27	95.86	10^{-10}	10^{-5}	ND
BTS1	-0.279	-0.967	0.9986	90.27	88.05	92.52	10^{-11}	10^{-5}	39.8
CrtE	-0.279	-0.751	0.9978	90.20	87.04	93.40	10^{-10}	10^{-5}	ND
CrtI	-0.295	-0.332	0.9980	97.44	94.15	100.79	10^{-10}	10^{-5}	ND
CrtYB	-0.281	-0.857	0.9981	90.91	88.36	93.50	10^{-11}	10^{-5}	ND
TAF10	-0.288	-0.772	0.9988	94.28	92.04	96.55	10^{-10}	10^{-5}	ND
UBC6	-0.286	-0.625	0.9988	93.31	91.07	95.58	10^{-10}	10^{-5}	38.76
ALG9	-0.282	-0.630	0.9983	91.62	88.69	94.60	10^{-10}	10^{-5}	37.06
ACT1	-0.281	-0.962	0.9992	91.17	89.44	92.91	10^{-11}	10^{-5}	37.57

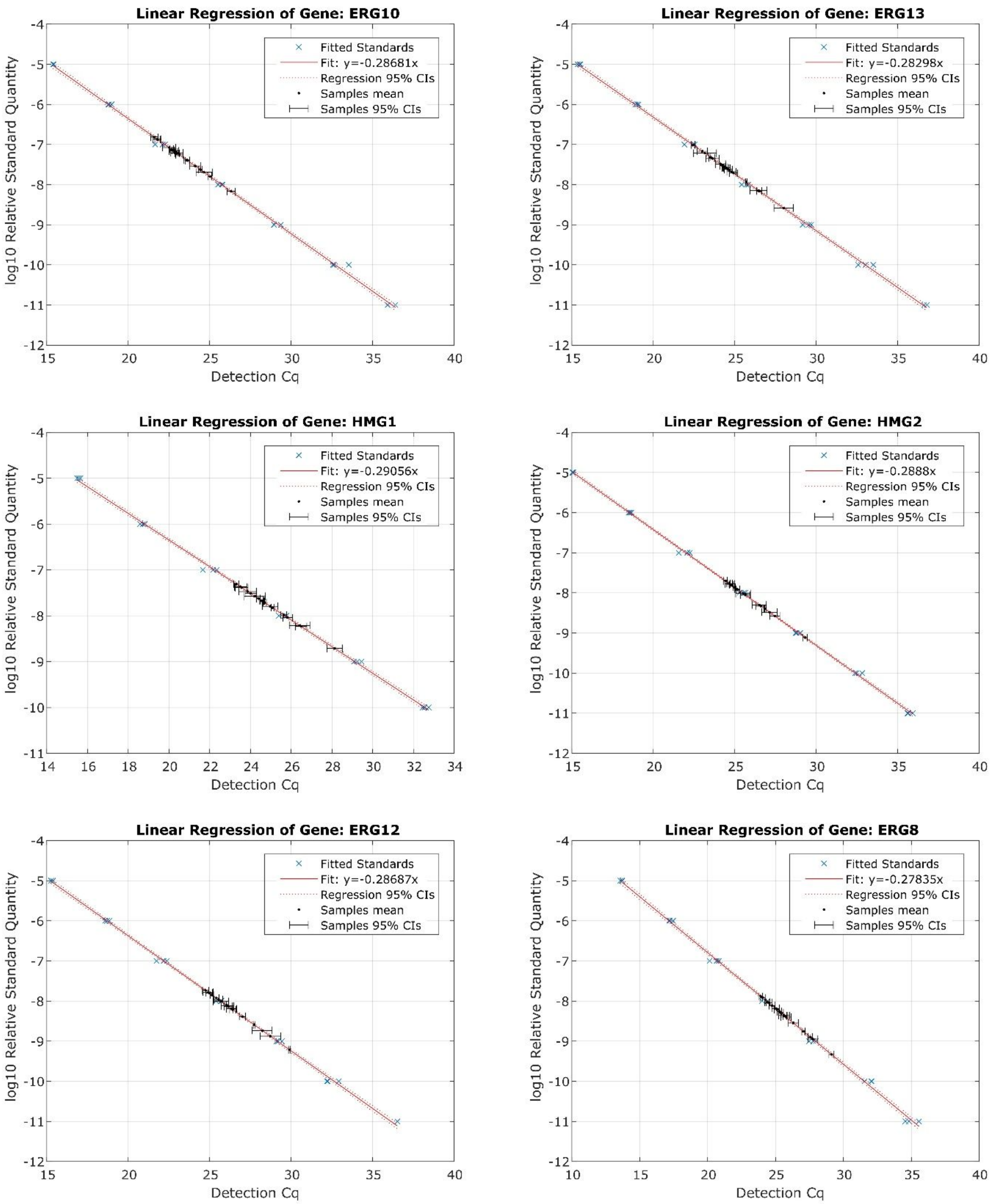

Figure S16. Linear regressions of the standard curves and mean C_q values of the samples for genes ERG10, ERG13, HMG1, HMG2, ERG12, and ERG8. 95% confidence regions for the regressions and technical replicates mean C_q values are included.

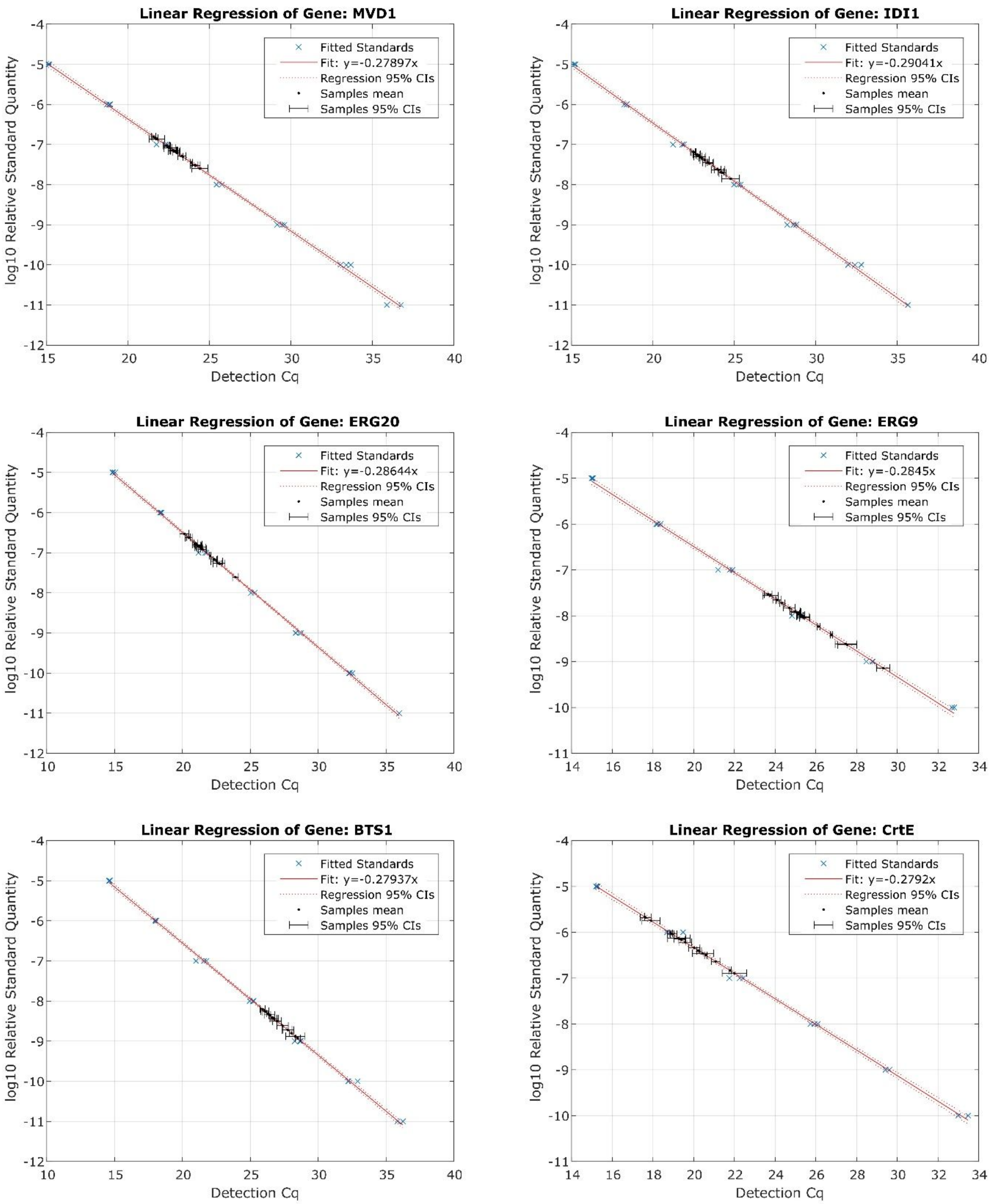

Figure S17. Linear regressions of the standard curves and mean C_q values of the samples for genes MVD1, IDI1, ERG20, ERG9, BTS1, and CrtE. 95% confidence regions for the regressions and technical replicates mean C_q values are included.

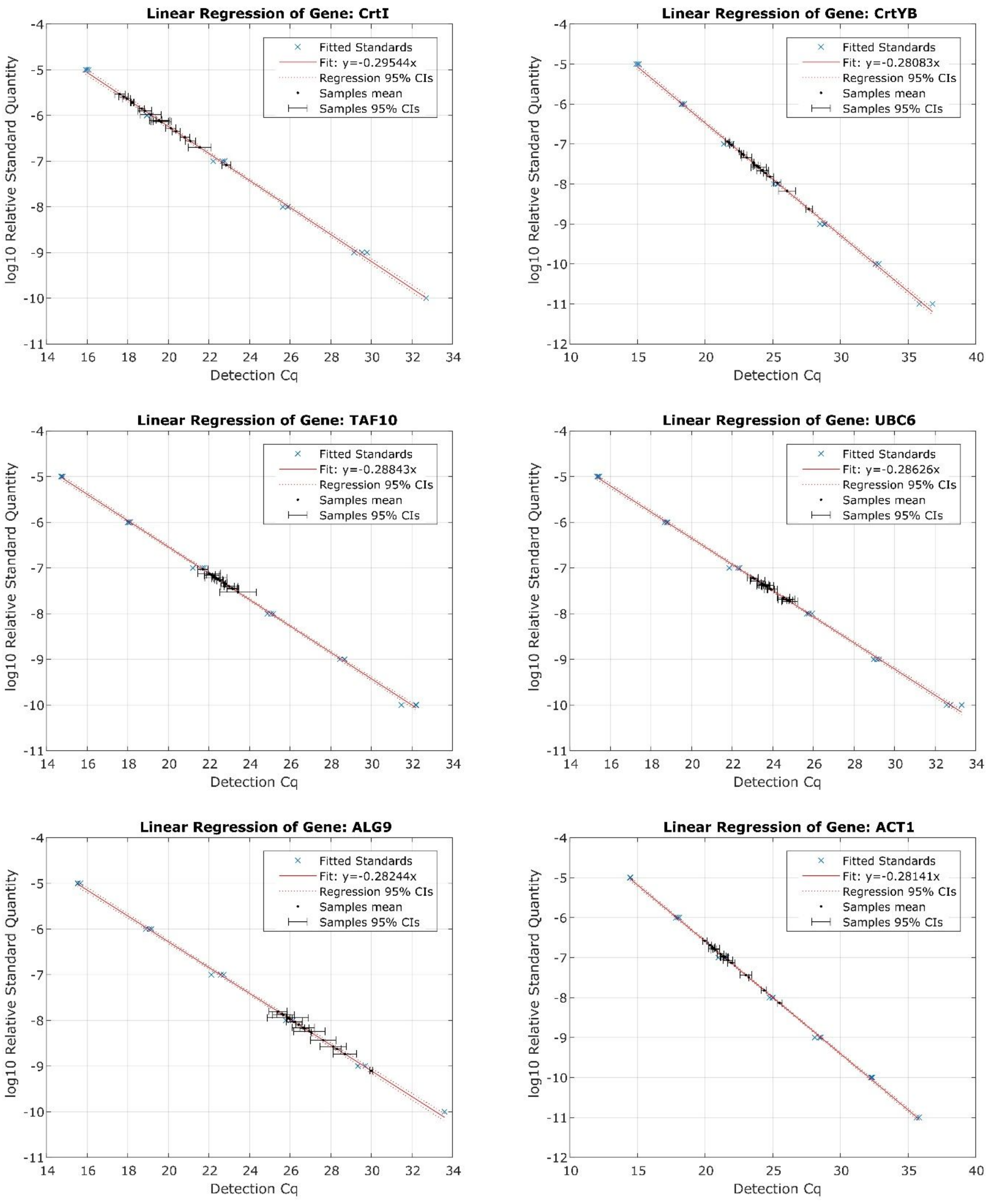

Figure S18. Linear regressions of the standard curves and mean C_q values of the samples for genes CrtI, CrtYB, TAF10, UBC6, ALG9, and ACT1. 95% confidence regions for the regressions and technical replicates mean C_q values are included.

S4. Experimental estimation of metabolic rates

The estimated parametric distributions of (gDCW/L)/OD600nm conversion factors, dilution rates, chemostat OD600nm, metabolite concentrations, were employed for metabolic flux calculations. Metabolite measurements obtained from high-performance liquid chromatography were checked for outliers using the median criterion included in MATLAB R2021a. Measurements considered as outliers were not considered in further analysis. The selected measurements were used to calculate the mean and standard error of metabolite concentrations in the feed media and the chemostat cultivations. It was assumed that the mean metabolite concentrations follow a t-distribution.

The mean metabolic fluxes were estimated by Monte Carlo simulations using MATLAB R2021a[13]. Values were sampled from the *t*-distributions defined for each variable. A total of 1 million simulated particles were used to estimate the distribution of the mean metabolic fluxes. This enabled the inclusion of the main sources of uncertainty to define appropriate ranges for the sampling of feasible kinetic models. The 95% confidence intervals were estimated as the 2.5 and 97.5 percentiles of the mean metabolic flux populations generated. Equation S1 shows the calculation formula of each metabolic flux sample. r is the metabolic flux rate, C_{in} the metabolite concentration in the feed, C the metabolite concentration in the chemostat cultivation, D the dilution rate, X the biomass measurements in OD600nm, and F the conversion factor for biomass concentration.

$$r\left[\frac{mmol}{gDCWh}\right] = \frac{\left(\left(C\left[\frac{mmol}{L}\right] - C_{in}\left[\frac{mmol}{L}\right]\right) * D\left[\frac{1}{h}\right]\right)}{X\,[OD600nm] * F\left[\frac{(gDCW/L)}{OD600nm}\right]} \qquad \text{(S1)}$$

Metabolic fluxes were transformed from mmol/gDCW/h to mmol/L/h for their use in the GRASP platform. Estimated values for cell density and moisture content were obtained from literature. It was assumed that mean cell density follows a normal distribution with an average of 1.1029g/ml and a standard deviation of 0.0026g/ml[23]. Whereas the mean moisture content is represented by a normal distribution with an average of 1.525g/gDCW and a standard deviation of 0.004g/gDCW[24]. As for the other variables, 1 million values were sampled from their distributions to estimate the mean metabolic flux distributions in mmol/L/h. Confidence intervales were also estimated using

the percentiles of the populations. This enabled the estimation of fluxes SK_lyc and SK_b_car for the kinetic model. Equation S2 shows the transformation formula of each flux sample. ρ is the cell density and M is the moisture content.

$$r\left[\frac{mmol}{L*h}\right] = \frac{r\left[\frac{mmol}{gDCWh}\right] * \rho\left[\frac{g}{L}\right]}{M\left[\frac{g}{gDCW}\right]} \qquad \text{(S2)}$$

The ERG9b fluxes were estimated by flux variability analysis (FVA)[25]. The genome-scale model (GEM) presented in the study by López et al. [26] was used. Like in that study, it was assumed that in stationary-state, all the compounds downstream of squalene in the GEM are not exchanged with the extracellular media. The range of exchange fluxes were defined according to growth conditions, the 95% confidence intervals of metabolic fluxes in mmol/gDCW/h, and the 95% confidence intervals of the growth rates. However, the ERG9b ('SQLS' reaction in the GEM) flux ranges only depended on the growth rate according to the results of the GEM. It was supposed that the average of the computed SQLS ranges is the mean ERG9b metabolic flux. Furthermore, it was considered that the mean ERG9b metabolic flux follows a t-distribution. It has the same freedom degrees as the t-distribution of dilution rates with its 95% confidence intervals coinciding with the ranges of the SQLS reaction. The ERG9b fluxes were transformed to mmol/L/h applying the same Monte Carlo procedure employed for SK_b_car and SK_lyc fluxes.

S5. Estimation of relative enzyme abundances

Relative transcript abundances were utilized as approximations for relative enzyme abundances. The Common Base method with additional steps was performed to calculate the relative mean mRNA concentrations and their 95% confidence intervals for each gene in the pathway. The details of the method are explained in the study of Ganger[27], and only the additional steps applied will be explained. All calculations were performed in the environment MATLAB R2021a[13]

β-car2 and β-car3 strains (non-reference conditions) were relativized to the strain β-car4 (reference condition) grown at the same dilution rate. All transcript measurements of a metabolic condition were relativized using the same reference genes, but not all non-reference conditions used the same set of reference genes. The selection of reference genes was based on the minimization of the largest confidence interval of the relative transcript abundance in a non-reference condition. To achieve this, all possible combinations of the reference genes TAF10, UBC6, ALG9, and UBC6 were tested for normalization. Therefore, a total of 15 reference gene sets were employed to perform the Common Base Method for each relativization by separate. In all cases, the non-reference and the reference conditions used the same set of reference genes. The efficiency-weighted delta quantification cycle (ΔC_q) of both conditions were checked for outliers using the median criterion included in MATLAB R2021a. The extraction replicates considered as outliers were discarded. Then the difference between the remaining ΔC_q of both conditions was estimated using a non-paired t-test of unequal variances. It was non-paired since extraction replicates are not correlated between different metabolic conditions. The estimated difference represents the mean efficiency-weighted delta delta quantification cycle ($\Delta\Delta C_q$) and its confidence intervals. Then the means and confidence intervals were transformed to estimated expression ratios ($10^{-\Delta\Delta Cq}$). The set of reference genes that achieved the minimum largest range confidence interval for all the genes was selected to relativize the corresponding non-reference condition. This can be represented by Equation S3:

$$\min_{\forall R}\left(\max_{\forall GOI}\left(Upper\ 95\%\ CI\ 10^{-\Delta\Delta \mathrm{Cq}}_{t,GOI,R} - Lower\ 95\%C\ I\ 10^{-\Delta\Delta \mathrm{Cq}}_{t,GOI,R}\right)\right) \qquad \text{(S3)}$$

Where R is one of the possible combinations of reference genes employed, GOI each gene of interest included in the pathway, and the subscript t is the non-reference condition that is being

relativized. Table S18 indicates which reference genes were used when relativizing each non-reference condition.

Table S18. Reference genes employed to relativize non-reference conditions.

	Growth rate (1/h)	0.101		0.254	
	Strain	β-car2	β-car3	β-car2	β-car3
Reference Genes	TAF10	X	✓	✓	✓
	UBC6	✓	X	✓	✓
	ALG9	✓	✓	✓	✓
	ACT1	✓	✓	✓	✓

S6. Determination of feasible concentration ranges for unbalanced metabolites

As no measurements were obtained for intracellular metabolites except for lycopene and β-carotene, feasible ranges were derived from literature. Balanced metabolites were not constrained since their concentrations can vary depending on the operating condition of the metabolic network. The metabolite concentration limits attempt to encompass the diversity of possible concentrations and were determined as a relaxed union of measurements observed in the bibliographical sources. Values presented in literature as concentration per dry biomass were transformed to concentration per volume by employing the assumed average cell density and mean moisture content previously reported. The ranges and their references are included in Table S19.

Table S19. Unbalanced metabolite estimated concentration ranges. Minimum and maximum values for the kinetic models are included for the unbalanced metabolites which ranges were constrained based on the cited references.

Metabolite	min (M)	max (M)	References
accoa	3.5.E-09	2.2.E-03	[28–31]
coa	3.4.E-08	1.7.E-03	[30,31]
nadph	1.0.E-11	2.0.E-02	[30–35]
nadp	2.7.E-09	5.6.E-03	[30–35]
atp	1.3.E-04	1.1.E-01	[31,32,36–46]
adp	4.3.E-05	3.3.E-01	[31,36–40,42–46]
pi	4.3.E-03	7.4.E-01	[39,45]
fad	4.3.E-07	8.7.E-04	[30,31]

S7. Computational implementation

Both sampling and model ensemble simulations required the use of parallel computing in the Unix-based HPC at the School of Engineering of the Pontificia Universidad Católica de Chile. The information of each computation node in the cluster is presented in Table S20. The number of cores destined for each task varied depending on the computational demand and the amount/type of processors available at the time of the processes. The computational resources employed for the sampling and simulation of detailed ensembles are included in Table S21.

Table S20. Information of the nodes utilized for the computation of detailed structure ensemble models.

Node	Processors	Number of processors	Cores per processor	Total cores	RAM (GB)
n1	2 AMD EPYC 7702 3100 MHz	2	64	128	512
n2	AMD EPYC 7643 3900 MHz	2	48	96	256
n3	AMD EPYC 9554 3750 MHz	2	64	128	256
n11	AMD EPYC 7662 3900 MHz	2	64	128	512

Table S21. Nodes, cores utilized and approximate required time for the completion of tasks performed for the detailed structure ensemble models.

Task	Growth rate (h^{-1})	Node used	Cores employed	Approximate required time
Sampling and rejection	0.101	n2	48	40 days
Sampling and rejection	0.254	n3	32	30 days
First relative enzyme abundance step simulations	0.101	n3	16	4.3 days
First relative enzyme abundance step simulations	0.254	n2	16	4 days
First relative metabolite concentration step simulations	0.101	n3	16	1.7 days
First relative metabolite concentration step simulations	0.254	n1	16	1.8 days
tHMG1 simulations	0.101	n11	16	1.1 days
tHMG1 simulations	0.254	n11	16	0.9 days
Second relative enzyme abundance step simulations	0.101	n2	16	4.1 days
Second relative enzyme abundance step simulations	0.254	N3	16	4.6 days
Second relative metabolite concentration step simulations	0.101	n3	16	1.7 days
Second relative metabolite concentration step simulations	0.254	n1	16	2.3 days

References

(1) Kornblatt, J. A.; Rudney, H. Two Forms of Acetoacetyl Coenzyme A Thiolase in Yeast. *Journal of Biological Chemistry* **1971**, *246* (14), 4417–4423. https://doi.org/10.1016/S0021-9258(18)62028-9.

(2) Middleton, B. The Kinetic Mechanism of 3-Hydroxy-3-Methylglutaryl-Coenzyme A Synthase from Baker's Yeast. *Biochemical Journal* **1972**, *126* (1), 35–47. https://doi.org/10.1042/bj1260035.

(3) Friesen, J. A.; Rodwell, V. W. The 3-Hydroxy-3-Methylglutaryl Coenzyme-A (HMG-CoA) Reductases. *Genome Biol* **2004**, *5* (248). https://doi.org/https://doi.org/10.1186/gb-2004-5-11-248.

(4) Schulte, A. E.; van der Heijden, R.; Verpoorte, R. Purification and Characterization of Mevalonate Kinase from Suspension-Cultured Cells of Catharanthus Roseus (L.) G. Don. *Arch Biochem Biophys* **2000**, *378* (2), 287–298. https://doi.org/10.1006/abbi.2000.1779.

(5) Pilloff, D.; Dabovic, K.; Romanowski, M. J.; Bonanno, J. B.; Doherty, M.; Burley, S. K.; Leyh, T. S. The Kinetic Mechanism of Phosphomevalonate Kinase. *Journal of Biological Chemistry* **2003**, *278* (7), 4510–4515. https://doi.org/10.1074/jbc.M210551200.

(6) Jabalquinto, A. M.; Cardemil, E. Substrate Binding Order in Mevalonate 5-Diphosphate Decarboxylase from Chicken Liver. *Biochimica et Biophysica Acta (BBA) - Protein Structure and Molecular Enzymology* **1989**, *996* (3), 257–259. https://doi.org/10.1016/0167-4838(89)90256-2.

(7) Berthelot, K.; Estevez, Y.; Deffieux, A.; Peruch, F. Isopentenyl Diphosphate Isomerase: A Checkpoint to Isoprenoid Biosynthesis. *Biochimie* **2012**, *94* (8), 1621–1634. https://doi.org/10.1016/j.biochi.2012.03.021.

(8) Liang, P.-H. Reaction Kinetics, Catalytic Mechanisms, Conformational Changes, and Inhibitor Design for Prenyltransferases. *Biochemistry* **2009**, *48* (28), 6562–6570. https://doi.org/10.1021/bi900371p.

(9) Tachibana, A.; Tanaka, T.; Taniguchi, M.; Oi, S. Potassium-Stimulating Mechanism of Geranylgeranyl Diphosphate Synthase of Methanobacterium Thermoformicicum SF-4. *The Journal of Biochemistry* **1993**, *114* (3), 389–392. https://doi.org/10.1093/oxfordjournals.jbchem.a124186.

(10) Liu, C.-I.; Jeng, W.-Y.; Chang, W.-J.; Shih, M.-F.; Ko, T.-P.; Wang, A. H.-J. Structural Insights into the Catalytic Mechanism of Human Squalene Synthase. *Acta Crystallogr D Biol Crystallogr* **2014**, *70* (2), 231–241. https://doi.org/10.1107/S1399004713026230.

(11) Schaub, P.; Yu, Q.; Gemmecker, S.; Poussin-Courmontagne, P.; Mailliot, J.; McEwen, A. G.; Ghisla, S.; Al-Babili, S.; Cavarelli, J.; Beyer, P. On the Structure and Function of the Phytoene Desaturase CRTI from Pantoea Ananatis, a Membrane-Peripheral and FAD-Dependent Oxidase/Isomerase. *PLoS One* **2012**, *7* (6), e39550. https://doi.org/10.1371/journal.pone.0039550.

(12) Moise, A. R.; Al-Babili, S.; Wurtzel, E. T. Mechanistic Aspects of Carotenoid Biosynthesis. *Chemical Reviews*. January 8, 2014, pp 164–193. https://doi.org/10.1021/cr400106y.

(13) The MathWorks Inc. MATLAB . The MathWorks Inc.: Natick, Massachusetts 2021. https://www.mathworks.com (accessed 2024-07-30).

(14) Masek, T.; Vopalensky, V.; Suchomelova, P.; Pospisek, M. Denaturing RNA Electrophoresis in TAE Agarose Gels. *Anal Biochem* **2005**, *336* (1), 46–50. https://doi.org/10.1016/j.ab.2004.09.010.

(15) Teste, M. A.; Duquenne, M.; François, J. M.; Parrou, J. L. Validation of Reference Genes for Quantitative Expression Analysis by Real-Time RT-PCR in Saccharomyces Cerevisiae. *BMC Mol Biol* **2009**, *10*, 99. https://doi.org/10.1186/1471-2199-10-99.

(16) Liu, S.; Bai, M.; Zhou, J.; Jin, Z.; Xu, Y.; Yang, Q.; Zhou, J.; Zhang, S.; Mao, J. Analysis of Genes from Saccharomyces Cerevisiae HJ01 Participating in Aromatic Alcohols Biosynthesis during Huangjiu Fermentation. *LWT* **2022**, *154*. https://doi.org/10.1016/j.lwt.2021.112705.

(17) Cankorur-Cetinkaya, A.; Dereli, E.; Eraslan, S.; Karabekmez, E.; Dikicioglu, D.; Kirdar, B. A Novel Strategy for Selection and Validation of Reference Genes in Dynamic Multidimensional Experimental Design in Yeast. *PLoS One* **2012**, *7* (6). https://doi.org/10.1371/journal.pone.0038351.

(18) Del Aguila, E. M.; Dutra, M. B.; Silva, J. T.; Paschoalin, V. M. F. Comparing Protocols for Preparation of DNA-Free Total Yeast RNA Suitable for RT-PCR. *BMC Mol Biol* **2005**, *6*. https://doi.org/10.1186/1471-2199-6-9.

(19) Vaudano, E.; Noti, O.; Costantini, A.; Garcia-Moruno, E. Identification of Reference Genes Suitable for Normalization of RT-QPCR Expression Data in Saccharomyces Cerevisiae during Alcoholic Fermentation. *Biotechnol Lett* **2011**, *33* (8), 1593–1599. https://doi.org/10.1007/s10529-011-0603-y.

(20) Ye, J.; Coulouris, G.; Zaretskaya, I.; Cutcutache, I.; Rozen, S.; Madden, T. L. Primer-BLAST: A Tool to Design Target-Specific Primers for Polymerase Chain Reaction. *BMC Bioinformatics* **2012**, *13* (1), 134. https://doi.org/10.1186/1471-2105-13-134.

(21) Owczarzy, R.; Tataurov, A. V.; Wu, Y.; Manthey, J. A.; McQuisten, K. A.; Almabrazi, H. G.; Pedersen, K. F.; Lin, Y.; Garretson, J.; McEntaggart, N. O.; Sailor, C. A.; Dawson, R.

B.; Peek, A. S. IDT SciTools: A Suite for Analysis and Design of Nucleic Acid Oligomers. *Nucleic Acids Res* **2008**, *36* (Web Server issue). https://doi.org/10.1093/nar/gkn198.

(22) Benson, D. A.; Cavanaugh, M.; Clark, K.; Karsch-Mizrachi, I.; Lipman, D. J.; Ostell, J.; Sayers, E. W. GenBank. *Nucleic Acids Res* **2013**, *41* (D1). https://doi.org/10.1093/nar/gks1195.

(23) Bryan, A. K.; Goranov, A.; Amon, A.; Manalis, S. R. Measurement of Mass, Density, and Volume during the Cell Cycle of Yeast. *Proceedings of the National Academy of Sciences* **2010**, *107* (3), 999–1004. https://doi.org/10.1073/pnas.0901851107.

(24) Illmer, P.; Erlebach, C.; Schinner, F. A Practicable and Accurate Method to Differentiate between Intra- and Extracellular Water of Microbial Cells. *FEMS Microbiol Lett* **1999**, *178* (1), 135–139. https://doi.org/10.1111/j.1574-6968.1999.tb13769.x.

(25) Gudmundsson, S.; Thiele, I. Computationally Efficient Flux Variability Analysis. *BMC Bioinformatics* **2010**, *11* (1), 489. https://doi.org/10.1186/1471-2105-11-489.

(26) López, J.; Bustos, D.; Camilo, C.; Arenas, N.; Saa, P. A.; Agosin, E. Engineering Saccharomyces Cerevisiae for the Overproduction of β-Ionone and Its Precursor β-Carotene. *Front Bioeng Biotechnol* **2020**, *8*. https://doi.org/10.3389/fbioe.2020.578793.

(27) Ganger, M. T.; Dietz, G. D.; Ewing, S. J. A Common Base Method for Analysis of QPCR Data and the Application of Simple Blocking in QPCR Experiments. *BMC Bioinformatics* **2017**, *18* (1), 534. https://doi.org/10.1186/s12859-017-1949-5.

(28) Seker, T.; Møller, K.; Nielsen, J. Analysis of Acyl CoA Ester Intermediates of the Mevalonate Pathway in Saccharomyces Cerevisiae. *Appl Microbiol Biotechnol* **2005**, *67* (1), 119–124. https://doi.org/10.1007/s00253-004-1697-0.

(29) Lian, J.; Si, T.; Nair, N. U.; Zhao, H. Design and Construction of Acetyl-CoA Overproducing Saccharomyces Cerevisiae Strains. In *Food, Pharmaceutical and Bioengineering Division 2014 - Core Programming Area at the 2014 AIChE Annual Meeting*; American Institute of Chemical Engineers, 2014; Vol. 2, pp 750–760. https://doi.org/10.1016/j.ymben.2014.05.010.

(30) Kumar, K.; Bruheim, P. Large Dependency of Intracellular NAD and CoA Pools on Cultivation Conditions in Saccharomyces Cerevisiae. *BMC Res Notes* **2021**, *14* (1). https://doi.org/10.1186/s13104-021-05783-6.

(31) Kozak, B. U.; van Rossum, H. M.; Benjamin, K. R.; Wu, L.; Daran, J. M. G.; Pronk, J. T.; Van Maris, A. J. A. Replacement of the Saccharomyces Cerevisiae Acetyl-CoA Synthetases by Alternative Pathways for Cytosolic Acetyl-CoA Synthesis. *Metab Eng* **2014**, *21*, 46–59. https://doi.org/10.1016/j.ymben.2013.11.005.

(32) Lange, H. C.; Eman, M.; Van Zuijlen, G.; Visser, D.; Van Dam, J. C.; Frank, J.; De Teixeira Mattos, M. J.; Heijnen, J. J. Improved Rapid Sampling for in Vivo Kinetics of Intracellular Metabolites in Saccharomyces Cerevisiae. *Biotechnol Bioeng* **2001**, *75* (4), 406–415. https://doi.org/10.1002/bit.10048.

(33) Zhang, J.; Pierick, A. Ten; Van Rossum, H. M.; Maleki Seifar, R.; Ras, C.; Daran, J. M.; Heijnen, J. J.; Aljoscha Wahl, S. Determination of the Cytosolic NADPH/NADP Ratio in Saccharomyces Cerevisiae Using Shikimate Dehydrogenase as Sensor Reaction. *Sci Rep* **2015**, *5*. https://doi.org/10.1038/srep12846.

(34) Vaseghi, S.; Baumeister, A.; Rizzi, M.; Reuss, M. In VivoDynamics of the Pentose Phosphate Pathway InSaccharomyces Cerevisiae. *Metab Eng* **1999**, *1* (2), 128–140. https://doi.org/10.1006/mben.1998.0110.

(35) Moreira Dos Santos, M.; Raghevendran, V.; Kötter, P.; Olsson, L.; Nielsen, J. Manipulation of Malic Enzyme in Saccharomyces Cerevisiae for Increasing NADPH Production Capacity Aerobically in Different Cellular Compartments. *Metab Eng* **2004**, *6* (4), 352–363. https://doi.org/10.1016/j.ymben.2004.06.002.

(36) Larsson, C.; Nilsson, A.; Blomberg, A.; Gustafsson, L. Glycolytic Flux Is Conditionally Correlated with ATP Concentration in Saccharomyces Cerevisiae: A Chemostat Study under Carbon- or Nitrogen-Limiting Conditions. *J Bacteriol* **1997**, *179* (23), 7243–7250. https://doi.org/10.1128/jb.179.23.7243-7250.1997.

(37) Larsson, C.; Påhlman, I. L.; Gustafsson, L. The Importance of ATP as a Regulator of Glycolytic Flux in Saccharomyces Cerevisiae. *Yeast* **2000**, *16* (9), 797–809. https://doi.org/10.1002/1097-0061(20000630)16:9<797::AID-YEA553>3.0.CO;2-5.

(38) Wu, L.; Van Dam, J.; Schipper, D.; Kresnowati, M. T. A. P.; Proell, A. M.; Ras, C.; Van Winden, W. A.; Van Gulik, W. M.; Heijnen, J. J. Short-Term Metabolome Dynamics and Carbon, Electron, and ATP Balances in Chemostat-Grown Saccharomyces Cerevisiae CEN.PK 113-7D Following a Glucose Pulse. *Appl Environ Microbiol* **2006**, *72* (5), 3566–3577. https://doi.org/10.1128/AEM.72.5.3566-3577.2006.

(39) Canelas, A. B.; Van Gulik, W. M.; Heijnen, J. J. Determination of the Cytosolic Free NAD/NADH Ratio in Saccharomyces Cerevisiae under Steady-State and Highly Dynamic Conditions. *Biotechnol Bioeng* **2008**, *100* (4), 734–743. https://doi.org/10.1002/bit.21813.

(40) Theobald, U.; Mailinger, W.; Baltes, M.; Rizzi, M.; Reuss, M. In Vivo Analysis of Metabolic Dynamics in Saccharomyces Cerevisiae: I. Experimental Observations. *Biotechnol Bioeng* **1997**, *55* (2), 305–316. https://doi.org/10.1002/(SICI)1097-0290(19970720)55:2<305::AID-BIT8>3.0.CO;2-M.

(41) Visser, D.; Van Zuylen, G. A.; Van Dam, J. C.; Eman, M. R.; Pröll, A.; Ras, C.; Wu, L.; Van Gulik, W. M.; Heijnen, J. J. Analysis of in Vivo Kinetics of Glycolysis in Aerobic Saccharomyces Cerevisiae by Application of Glucose and Ethanol Pulses. *Biotechnol Bioeng* **2004**, *88* (2), 157–167. https://doi.org/10.1002/bit.20235.

(42) Franco, C. M. M.; Smith, J. E.; Berry, D. R. Effect of Nitrogen and Phosphate on the Levels of Intermediates in Bakers' Yeast Grown in Continuous Culture. *Microbiology (N Y)* **1984**, *130* (10), 2465–2472. https://doi.org/10.1099/00221287-130-10-2465.

(43) Mashego, M. R.; van Gulik, W. M.; Vinke, J. L.; Visser, D.; Heijnen, J. J. In Vivo Kinetics with Rapid Perturbation Experiments in Saccharomyces Cerevisiae Using a Second-Generation BioScope. *Metab Eng* **2006**, *8* (4), 370–383. https://doi.org/10.1016/j.ymben.2006.02.002.

(44) Van Heerden, J. H.; Wortel, M. T.; Bruggeman, F. J.; Heijnen, J. J.; Bollen, Y. J. M.; Planqué, R.; Hulshof, J.; O'Toole, T. G.; Wahl, S. A.; Teusink, B. Lost in Transition: Start-up of Glycolysis Yields Subpopulations of Nongrowing Cells. *Science (1979)* **2014**, *343* (6174). https://doi.org/10.1126/science.1245114.

(45) Canelas, A. B.; Ras, C.; ten Pierick, A.; van Gulik, W. M.; Heijnen, J. J. An in Vivo Data-Driven Framework for Classification and Quantification of Enzyme Kinetics and Determination of Apparent Thermodynamic Data. *Metab Eng* **2011**, *13* (3), 294–306. https://doi.org/10.1016/j.ymben.2011.02.005.

(46) Kumar, K.; Venkatraman, V.; Bruheim, P. Adaptation of Central Metabolite Pools to Variations in Growth Rate and Cultivation Conditions in Saccharomyces Cerevisiae. *Microb Cell Fact* **2021**, *20* (1). https://doi.org/10.1186/s12934-021-01557-8.
